# Acquisition Order of Ras and p53 Gene Alterations Defines Distinct Adrenocortical Tumor Phenotypes

**DOI:** 10.1371/journal.pgen.1002700

**Published:** 2012-05-10

**Authors:** Maryline Herbet, Aude Salomon, Jean-Jacques Feige, Michaël Thomas

**Affiliations:** 1 Institut National de la Santé et de la Recherche, Unité 1036, Grenoble, France; 2 Commissariat à l'Énergie Atomique, Institut de Recherches en Technologies et Sciences pour le Vivant, Biologie du Cancer et de l'Infection, Grenoble, France; 3 Université Joseph Fourier-Grenoble I, Grenoble, France; Cincinnati Children's Hospital Medical Center, United States of America

## Abstract

Sporadic adrenocortical carcinomas (ACC) are rare endocrine neoplasms with a dismal prognosis. By contrast, benign tumors of the adrenal cortex are common in the general population. Whether benign tumors represent a separate entity or are in fact part of a process of tumor progression ultimately leading to an ACC is still an unresolved issue. To this end, we have developed a mouse model of tumor progression by successively transducing genes altered in adrenocortical tumors into normal adrenocortical cells. The introduction in different orders of the oncogenic allele of Ras (*H-Ras^G12V^*) and the mutant p53^DD^ that disrupts the p53 pathway yielded tumors displaying major differences in histological features, tumorigenicity, and metastatic behavior. Whereas the successive expression of Ras^G12V^ and p53^DD^ led to highly malignant tumors with metastatic behavior, reminiscent of those formed after the simultaneous introduction of p53^DD^ and Ras^G12V^, the reverse sequence gave rise only to benign tumors. Microarray profiling revealed that 157 genes related to cancer development and progression were differentially expressed. Of these genes, 40 were up-regulated and 117 were down-regulated in malignant cell populations as compared with benign cell populations. This is the first evidence-based observation that ACC development follows a multistage progression and that the tumor phenotype is directly influenced by the order of acquisition of genetic alterations.

## Author Summary

A sequential acquisition of genetic events is critical in tumorigenesis, and a dysregulation of a limited set of pathways has been demonstrated as sufficient to progressively transform normal cells into tumor cells in several human tissues. However, in the case of adrenocortical tumorigenesis, whether benign tumors represent a separate entity or are in fact part of a process of tumor progression leading ultimately to an adrenal carcinoma is still an unresolved issue. Moreover, the importance of the order in which these genetic events must occur to transform a cell has not been established. Here, we developed a tissue reconstruction model in mice that allows direct comparison of cells modified with sequential introduction of two genetic events. This revealed that adrenocortical tumor development follows a multistage progression and that the tumor phenotype, including histopathology and metastatic behavior, is directly influenced by the order of acquisition of genetic alterations.

## Introduction

Cancer is an heterogeneous disease. Thus, tumors in different organs display markedly different clinical behaviors and tumors that arise in a single tissue can even exhibit an array of pathologies, ranging from benign adenomas to highly invasive malignancies [1], [2]. The phenotypic diversity observed in neoplastic tumors has been generally ascribed to the deregulation of multiple signal transduction pathways. However, it remains unclear for most cancers which genetic alterations in a cell or group of cells play a causative role in tumor initiation and progression, and which ones represent bystanders with no selective advantage. Cellular transformation is a process where a normal cell accumulates mutations, as well as epigenetic changes, that activate oncogenes or down-regulate tumor suppressor genes to give rise to a clonal expansion of a subset of transformed cells, independently of both external and internal signals that normally control cell growth [3]. This general concept of multistage tumorigenesis has been demonstrated in the case of the colon cancer where distinct histological stages are directly correlated with genetic alterations in key tumor suppressors and oncogenes. Most adenomatous polyps of the colon, even though they are the precursors of invasive cancer, never actually progress to that stage [4], [5]. Thus, clinically the occurrence of benign tumors is much more frequent than carcinomas.

Sporadic adrenocortical carcinomas (ACCs) are rare endocrine neoplasms in humans, notorious for their aggressive behavior, metastatic potential and poor outcome [6] with an estimated worldwide annual incidence of 2 per million in adults [7]. By contrast, benign adrenocortical adenomas (ACAs) are rather common in the general population (present in 2.3% of persons at autopsy [8]). Moreover, the availability of high-resolution imaging modalities has resulted in an increase of the detection of adrenal masses of which cortical adenomas represent 52% of the surgically resected incidental tumors [9]. Whether ACAs represent a separate entity or are in fact part of a process of tumor progression leading to the emergence of ACC is still an open question, however these numbers are consistent with the hypothesis that only a very small fraction of ACAs may progress to cancer upon the accumulation of additional changes. Although the direct progression of benign adrenocortical tumors to malignant carcinomas has not been clearly demonstrated, the best evidence for a multistage adrenal tumorigenesis comes from two clinical cases where a localized tumor was found to be composed of a benign part surrounded by a malignant area [10], [11]. Progress into the elucidation of the genes and pathways involved in the pathogenesis of sporadic ACC has been slower than that for most other cancers, largely because of the rarity of this tumor [12]. Nevertheless it has been shown that *TP53* somatic mutations are present in about 30% of sporadic adult ACCs and almost never in ACAs [12] whereas, activating mutations of *N-Ras* gene have been observed in both benign and malignant adrenal cortical neoplasms with an incidence of 12.5% [13]. In addition, a suitable animal model for unraveling the role of a given genetic alteration and its possible cooperation with other gene defects in the pathogenesis of the disease has also been lacking.

We have previously determined that the sequential introductions of the catalytic subunit of the human telomerase, the simian virus 40 large T (LT) and an oncogenic allele of Ras (*H-Ras^G12V^*) suffice to transform normal bovine adrenocortical (BAC) cells into tumorigenic cells, when transplanted beneath the kidney capsule of SCID mice [14]. Our data suggested that a limited number of genetic alterations cooperate to transform long-lived mammalian adrenocortical cells. We sought now to develop an *in vivo* system for the neoplastic transformation of primary BAC cells in order to reveal a minimal set of genes that had been recognized to be altered in human adrenocortical tumors (ACT) and to study the influence of each of these genetic alterations taken separately on the pathogenesis of the disease.

Here, we report that the simultaneous disruption of the p53 pathway by using a truncated form of the protein, p53^DD^, which acts as a dominant-negative [15] and the Ras pathway through the stable expression of an active Ras protein (H-Ras^G12V^) [16] is sufficient to transform normal BAC cells into a tumorigenic state. Strikingly, we show, using our *in vivo* tissue reconstruction model, that the order of acquisition of genetic mutations is a critical determinant in the outcome of tumor development and aggressiveness.

## Results

### Expression of Ras^G12V^ and p53^DD^ in BAC cells alters their growth properties in culture

**Figure 1:**
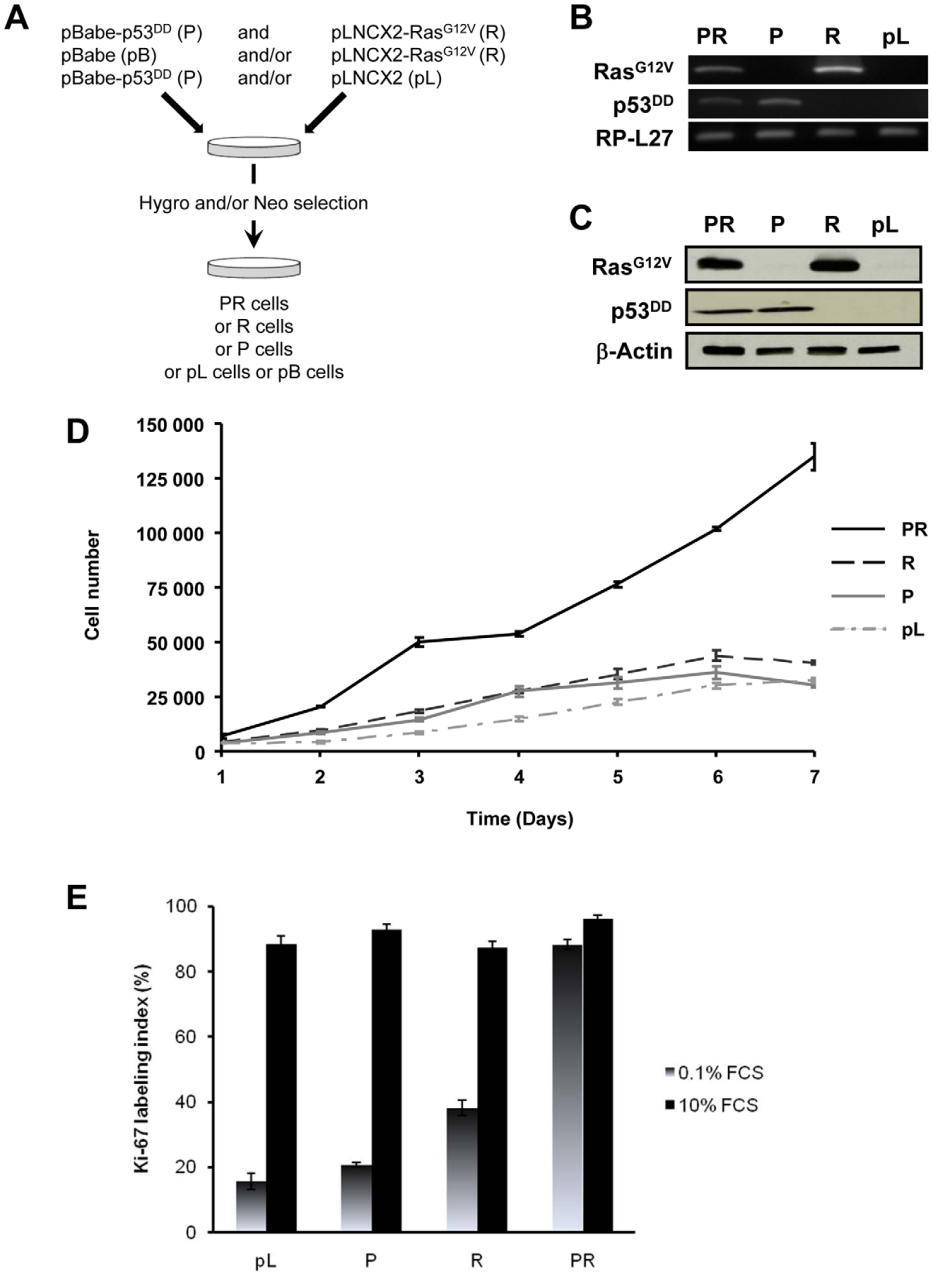
*In vitro* characterization of BAC cells transduced simultaneously with p53^DD^ and Ras^G12V^. A, summary of the experimental design for the generation of stably infected cell populations. B, detection of Ras^G12V^ and p53^DD^ by RT-PCR in adrenocortical PR, P, R and pL cells. Total RP-L27 acts as loading control. C, Confirmation of protein expression by immunoblotting of PR, R, P and pL cells. Actin served as a loading control. D, growth curves of PR, R, P and pL cells. The *in vitro* cell proliferation rates were obtained by counting cells from triplicate cell culture dishes every day. *Points*, mean; *bars*, SD. E, asynchronous populations of BAC cells were cultured in normal culture medium or in defined medium containing 0.1% FCS. The percentage of cells engaged into the cell cycle was determined by measuring the Ki-67 labeling index. *Bars*, SD.

The primary BAC cells were infected simultaneously with two replication-defective amphotropic retroviruses based on Moloney murine leukemia virus (MoMLV) expressing either *p53^DD^* [17] or *H-Ras^G12V^* [18], each encoding a drug selection marker, hygromycin and neomycin, respectively. Following infection, the cells were selected for 7 days by supplementing the culture medium with both antibiotics. At the end of the selection process, we established a polyclonal population termed p53^DD^/Ras^G12V^ (PR) cells (Figure 1A). Two parallel cultures of primary BAC cells were infected simultaneously either with a retrovirus expressing *p53^DD^* (P) and a control pLNCX2 (pL) retrovirus, or with a retrovirus expressing *H-Ras^G12V^* (R) and a control pBabe-Hygro (pB) retrovirus. Thus, we generated two control populations termed P and R, respectively (Figure 1A).

We first confirmed that the three polyclonal BAC cell populations transduced with p53^DD^ (P), Ras^G12V^ (R) or both p53^DD^ and Ras^G12V^ (PR) expressed the desired transgenes (Figure 1B). Then the cells were assayed for the expression of the desired transgenes by immunoblot analysis. We found that the resulting polyclonal cell populations expressed similar amounts of Ras^G12V^ and p53^DD^ proteins (Figure 1C) The replication of pL, R and P cells ceased at high density suggesting that these cells were still sensitive to contact inhibition (Figure 1D), a regulatory mechanism through which cells enter a stage of reversible G1 arrest [19]. On the contrary, PR cells did not demonstrate any decrease in cell proliferation at high cell density (Figure 1D) and formed multilayered foci in culture (data not shown), a phenomenon commonly associated with malignant transformation [19]. Thus, infection of adrenocortical cells with the combination of p53^DD^ and Ras^G12V^ dramatically increased the proliferation rate in comparison to infection with either p53^DD^ or Ras^G12V^ alone (Figure 1D). We also studied the proliferation by determining the percentage of Ki-67 positive cells in each cell population. In serum-supplemented medium, each of these populations displayed a similar percentage of cells engaged in the cell cycle (Figure 1E). However, in the absence of serum, only cells transduced with Ras^G12V^ and p53^DD^ proliferated independently from extrinsic mitogens. Conversely, pL and P cells required mitogens for their proliferation, whereas R cells exhibited a reduced dependence to growth factors (Figure 1E). Therefore, in cells with defective p53 signaling, oncogenic Ras is able to partially substitute for a mitogenic signal. Finally, the PR cell population and the two control cell populations P and R were seeded in soft agar to assay for anchorage-independent growth. Whereas expression of p53^DD^ was unable to support anchorage-independent growth of adrenocortical cells, cells expressing Ras^G12V^ formed small abortive colonies characteristic of transit-amplifying cells (Table 1). Only the expression of both p53^DD^ and Ras^G12V^ led to robust cell growth in soft agar (Table 1).

**Table 1:** Anchorage-independent growth of the adrenocortical cells expressing the indicated transgenes.

Soft Agar assay	
PR	191±5
P	0
R	47±3
R+P	106±2
P+R	36±2

The number of soft agar colonies was determined 3 weeks after 5×10^3^ cells were seeded. Results are expressed as the mean ± SD of three determinations in three distinct experiments.

We thus concluded from these experiments that PR cells were transformed since they displayed all the *in vitro* characteristics ascribed to tumor cells, *i.e.* loss of contact inhibition in culture, proliferation in the absence of extrinsic mitogens and in the absence of anchorage.

### Rapid tumor induction by BAC cells expressing Ras^G12V^ and p53^DD^

Although mutation or overexpression of genes such as *TP53* and *Ras* are detected in human adrenocortical tumors [20], it is not known whether these genetic changes must occur in combination to induce tumor growth. We then wondered whether these two genetic changes are sufficient to endow BAC cells with the ability to form adrenocortical carcinoma *in vivo*.

**Figure 2:**
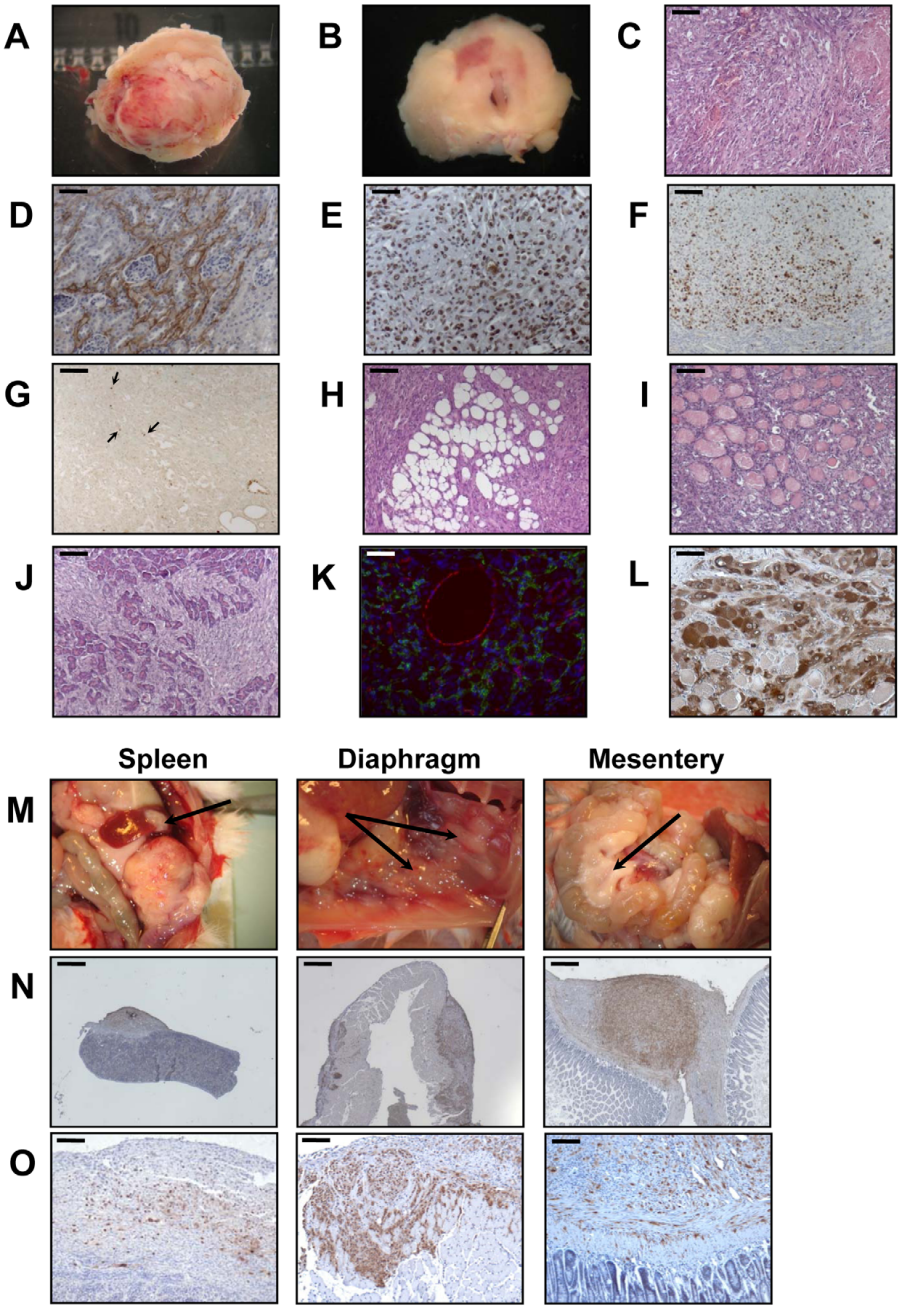
Malignant behavior of PR cells in the subrenal capsule assay. A, appearance of a representative entire tumor mass resulting from growth of transplanted PR cells at day 35. B, the tumors were cut transversally and photographed, together with the host animal kidney. C–G, H&E staining (bar, 100 µm) (C); Ras staining (bar, 50 µm) (D); p53 staining (bar, 50 µm) (E); Ki-67 staining (bar, 100 µm) (F); and TUNEL staining (arrows; bar, 100 µm) (G) of PR tumors. H–J, H&E coloration of tumor sections reveals invasion into adjacent tissues including adipose tissue (bar, 100 µm) (H); muscle (bar, 100 µm) (I); and pancreas (bar, 100 µm) (J). K, Double CD31 (red) and LYVE-1 (green) immunofluorescent staining of the tumors to detect vascular and lymphatic vessels, respectively (bar, 50 µm). L, 3βHSD immunostaining of the tumors to detect adrenocortical steroidogenic cells (bar, 50 µm). M–N, adrenocortical tumor spread to retroperitoneal organs. Arrows indicate PR cells metastases to the spleen, diaphragm and mesentery (M); Ras staining of metastases (bar, 400 µm) (N); p53 staining of metastases (bar, 100 µm) (O).

When the PR cells were transplanted beneath the subrenal capsule (SRC) of adrenalectomized SCID mice, the rate of tumor formation was 100% and no latency period was observable suggesting that the microenvironment provided by the SRC was favorable to tumor development. This was obvious on sections of the grafts taken on day 8, 14 and 21 (Figure S1). The transplanted PR cells produced continuously expanding tumor masses, which first protruded from the site of transplantation and finally destroyed the kidney (Figure S1A, S1B). Eight days after cell transplantation, the xenografts formed a solid tissue structure on the kidney surface (Figure S1B). Invasive characteristics of the tumors were evidenced by day 14, as they infiltrated the adjacent kidney parenchyma (Figure S1B, S1C) and by day 21, the neighboring tissues, skeletal muscle and adipose tissue (Figure S1D, S1E). Ultimately, by day 35, the kidney was destroyed (Figure 2A, 2B) and the adjacent tissues and organs (fat, muscle and pancreas) were invaded (Figure 2H–2J). The tumors were poorly differentiated carcinomas composed of eosinophilic cells with high nuclear grade, high mitotic activity and prominent nucleoli. Necrosis, a typical histopathological marker of malignancy was commonly observed in tumors at day 35 (Figure 2C). Rare apoptotic cells were detected in these tumors (Figure 2G). Examination of Ki-67 expression showed that the cells had a very high proliferation rate which was sustained over time (Figure S1C; Figure 2F and Table 2). Ras and p53 antibodies showed strong staining throughout the tumor, confirming the long term expression of the transgenes (Figure S1D, S1E; Figure 2D, 2E).

**Table 2:** Proliferation in tissues formed following transplantations of 2×10^6^ cells expressing the indicated transgenes either beneath the kidney capsule.

Site of implantation	Implanted cells	Days post-implantation	Ki-67 labeling index (%)
	pL	35	3.51±1.04
	P	35	4.57±1.21
	R	35	18.72±3.25
Subrenal capsule	PR	8	56.75±9.30
	PR	14	46.31±7.20
	PR	21	42.27±7.40
	PR	35	48.43±6.66

The labeling index which corresponds to the number of Ki-67^+^ cells per 100 adrenocortical cells was determined at various times after cell transplantation. Results are expressed as the mean of the counts of two non-consecutive tissue sections per condition +/− SD.

Metastases are responsible for 90% of deaths from solid tumors and arise following the spread of cancer cells from the primary site and the formation of new tumors in distant organs. The metastatic process comprises a series of steps including angiogenesis and lymphangiogenesis, which allow the tumor cells to escape the confines of the primary tumor [21]. Moreover, the formation of new blood vessels is an almost absolute requirement in the early development of tumors by providing oxygen and nutrients to the cells. We consistently observed a dense vascular network on the surface of the PR masses that was confirmed on tissue sections by immunofluorescence with an antibody against CD31 (Figure 2K). At day 35, it was clear from gross appearance that the primary tumor had spread to intraperitoneal organs. Metastatic sites included spleen, diaphragm, abdominal muscle and mesentery (Figure 2M). Most of the metastases grew on the surface of the organs (Figure 2M) and the cells forming the metastases were issued from the PR primary tumors as demonstrated by Ras and p53 expression (Figure 2N, 2O). As recent experimental studies and clinicopathological reports suggest that tumor lymphangiogenesis can promote tumor spread through the secretion of lymphangiogenic growth factors [21], [22], we investigated the presence of lymphatic vessels in primary tumors by immunofluorescence staining for LYVE-1 (lymphatic vessel endothelial hyaluronan receptor-1), a specific marker of lymphatic endothelial cells [23]. At the time when metastases were detected, numerous lymphatics were present in the tumor (Figure 2K), suggesting that the spread of tumor cells might occur through *de novo* development of a lymphatic network.

Adrenalectomized animals bearing transplanted cells lived well until they are sacrificed, demonstrating that the cells were functional and produced cortisol (data not shown). The mouse glucocorticoid, corticosterone, was replaced in plasma by the bovine glucocorticoid, cortisol. Cortisol levels gave an unambiguous measure of the function of the transplanted cells because mice lack expression of the steroid-17α-hydroxylase in the adrenal cortex, thus resulting in the biosynthesis of corticosterone rather than cortisol. In addition to cortisol production, we wanted to ascertain that the tumors and metastases were formed from BAC cells transformed by the transduction of p53^DD^ and Ras^G12V^ and not from cells such as fibroblasts or other stromal cells possibly contaminating the primary culture. Immunohistochemical analysis of the tumors for the expression of the steroid-converting enzyme 3-β-hydroxy-Δ5-steroid dehydrogenase/isomerase-1 type II (3βHSD) involved in cortisol biosynthesis showed that most if not all tumor cells were positively stained (Figure 2L), consistent with the steroidogenic origin of the initial cells.

The formation of malignant tumors by cells expressing only Ras^G12V^ and p53^DD^ was unexpected since it has been previously shown that adrenocortical cells require at least the ablation of two tumor suppressor genes (p53 and Rb through the expression of LT antigen) and the mutation of one oncogene (expression of Ras^G12V^) to undergo transformation [24]. To rule out the possibility that mutations other than the introduction of *Ras^G12V^* and *p53^DD^* occurred during the process of engineering the PR cells, we ensured to keep our population polyclonal, to reduce the period in culture for its generation as short as possible and to produce three independent other PR polyclonal populations from primary BAC cells. Following transplantation, tumors formed in 100% of the injected mice and all tumors were highly neoplastic, poorly differentiated and invaded the adjacent kidney and organs (data not shown). Since it is unlikely that all three polyclonal cell populations have acquired the same mutation that was essential for tumorigenesis, we can conclude that the malignant potential of the cell population is a property of transduced PR cells in general rather than the result of an overgrowth of a minor subpopulation. So, the simultaneous alteration of both p53 and Ras pathways is sufficient to fully transform primary BAC cells and form metastatic ACC when transplanted beneath the SRC of mice.

### p53^DD^or Ras^G12V^ alone are not sufficient to transform BAC cells into a tumorigenic state

**Figure 3:**
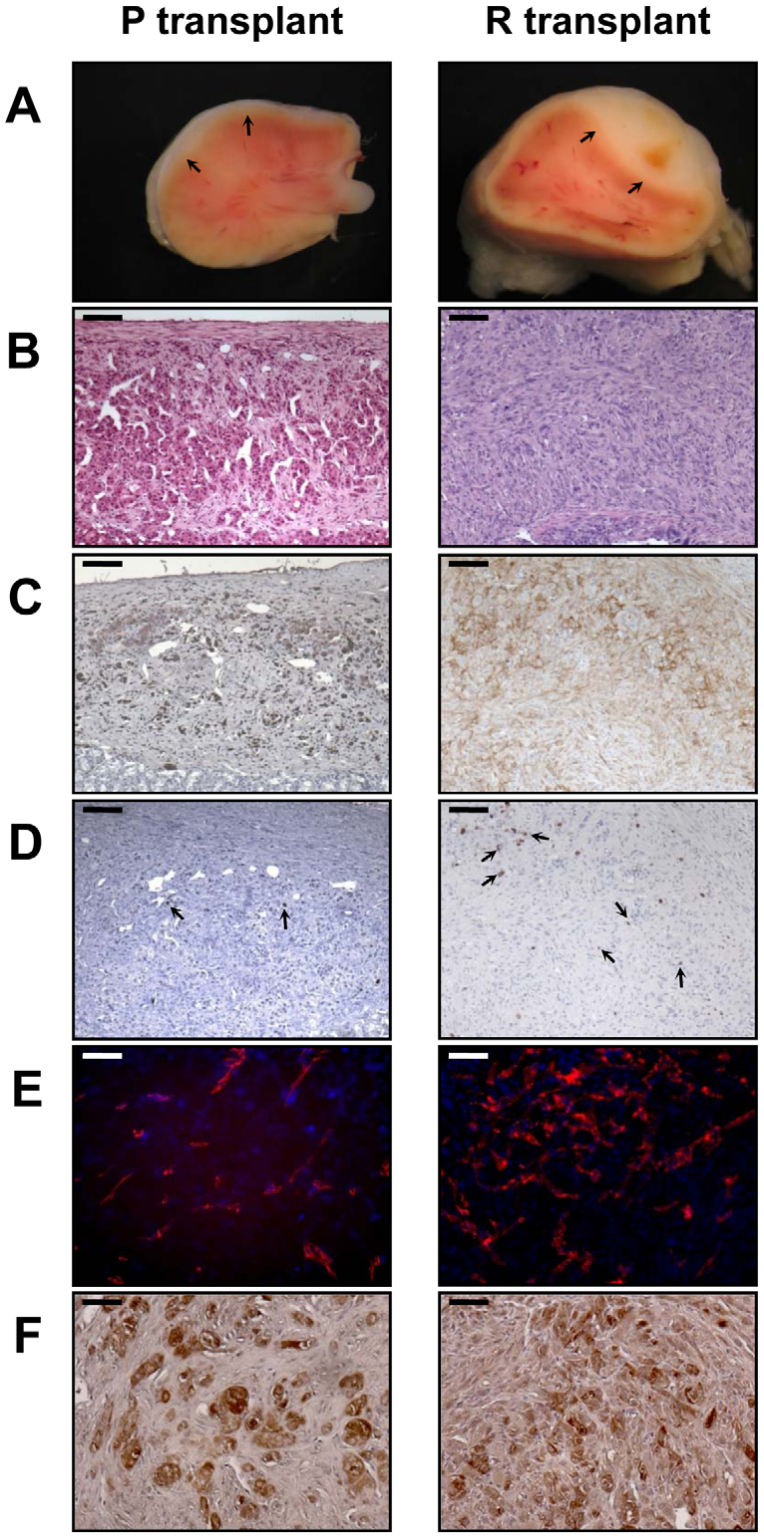
Characterization of tissues formed from P or R cell transplantation at day 35. 2×10^6^ P and R cells were transplanted beneath the kidney capsule of mice. The tissues found to have resulted from growth of the transplanted cells (arrows) were cut transversally and photographed, together with the host kidney (A). H&E stained sections (bar, 100 µm) (B); immunostained for p53 expression in P transplant or Ras expression in R transplant (bar, 100 µm) (C); assayed for Ki-67^+^ cells (arrows, bar, 100 µm) (D); double immunofluorescent stained for CD31 (red) and LYVE-1 (no green fluorescence detected) (bar, 50 µm) (E); and immunostained for 3βHSD (bar, 50 µm) (F).

**Figure 4:**
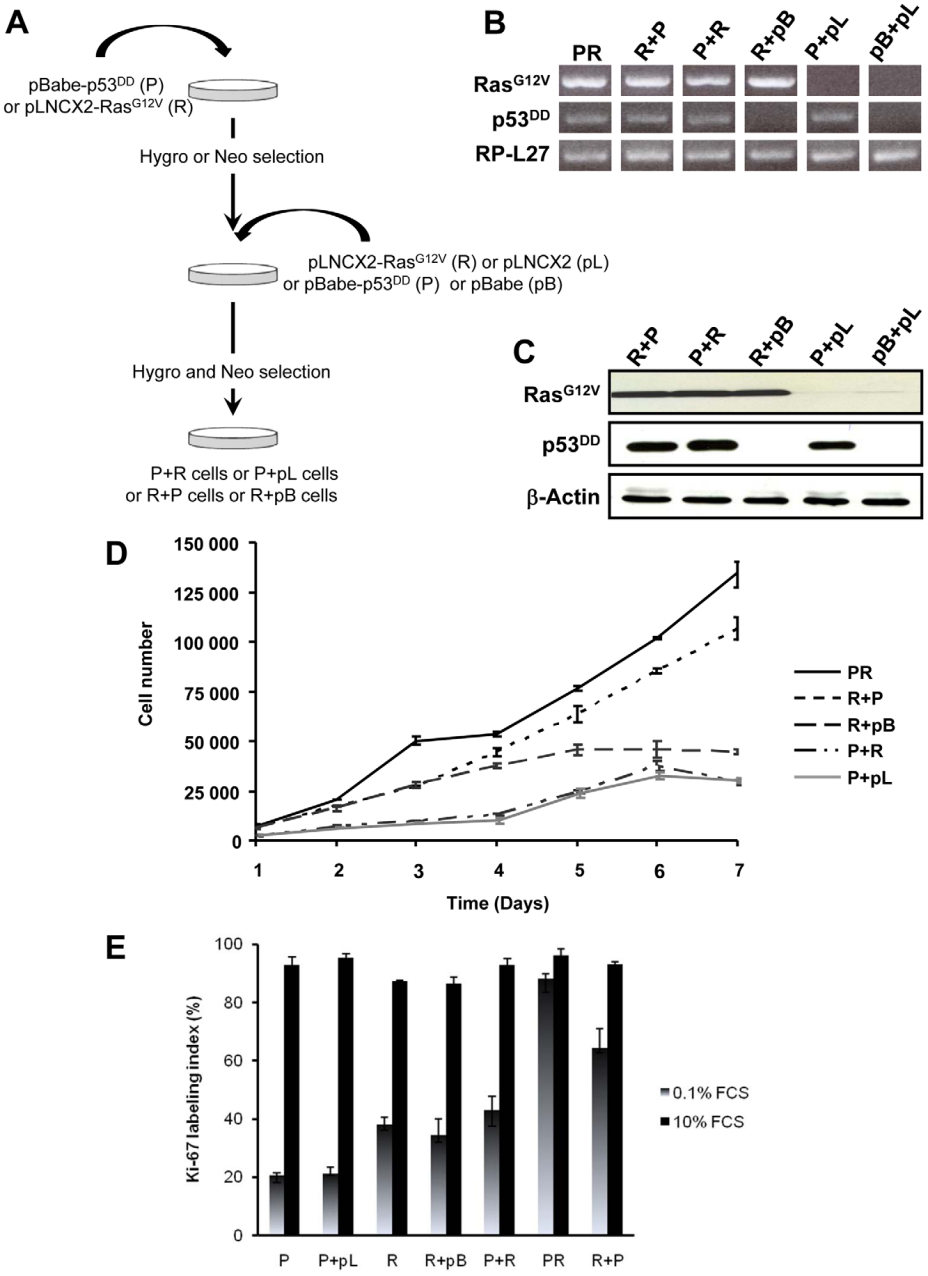
*In vitro* characterization of BAC cells transduced with p53^DD^ and Ras^G12V^ in succession and in distinct orders. A, summary of the experimental design for the generation of stably infected cell populations. B, detection of Ras^G12V^ and p53^DD^ by RT-PCR in BAC cells transduced with the indicated transgenes. Total RP-L27 acts as loading control. C, Confirmation of protein expression by immunoblotting of R+P, P+R, R+pB, P+pL and pB+pL cells. Actin served as a loading control D, growth curves of R+P, P+R, R+pB and P+pL cells. The *in vitro* cell proliferation rates were obtained by counting cells from triplicates cell culture dishes every day. For comparison, PR growth curve has been plotted on the same graph. *Points*, mean; *bars*, SD. E, asynchronous populations of P+pL, P+R, R+pB, and R+P cells were cultured in complete culture medium or in medium containing 0.1% FCS. The percentage of cells engaged into the cell cycle was determined by measuring the Ki-67 labeling index. For comparison, P, R and PR Ki-67 labeling index have been plotted on the same graph. *Bars*, SD.

Next, we analyzed the phenotype of tissues resulting from implantation of cells that had been singly transduced with *p53^DD^* or *Ras^G12V^* retroviruses. Following transplantation, P cells formed a small tissue spread between the kidney parenchyma and the capsule (Figure 3A). P tissue presented a uniform structure of regular eosinophilic adrenocortical cells without invasion in the renal parenchyma (Figure 3B). Examination of Ki-67 expression in tissue sections showed that the transplanted cells had a low proliferation rate (4.6%, Figure 3D; Table 2). In contrast, R cells gave rise to a voluminous tissue with no sign of invasion (Figure 3A). The R cells had formed an heterogeneous benign expanding tumor with an irregular architecture, cellular pleomorphism and some nuclear atypia (Figure 3B). We confirmed by p53 and Ras expression that these phenotypes were due to the expression of the transgenes (Figure 3C). The proliferation rate of the R cells in the transplants was intermediate between that measured in P tissues and in PR tissues (18.7%, Figure 4D; Table 2). Both P and R tissues were vascularized and interestingly, no lymphatics were detected consistent with the absence of the occurrence of metastases in the recipient mice (Figure 3E). Finally, the P and R tissues were functional as probed by cortisol production (data not shown) and by the detection of 3βHSD (Figure 3F), consistent as described above with the adrenal origin of the initial cells.

### The order of acquisition of genetic alterations is a critical determinant of the tumor phenotype

Simultaneous activation of the Ras oncogene and inactivation of the p53 tumor suppressor deregulate the transcriptional programs and confer PR adrenocortical cells a tumorigenic potential when transplanted into mice. However, the exact importance of the order of acquisition of these genetic events on the tumor phenotype has not yet been clearly established. It was proposed that multiple alternative genetic pathways may lead to the formation of a primary tumor and that the characteristics of a tumor may vary as a function of the activated pathway [25]. To address this important issue, primary cells were successively transduced with two retroviruses, allowing between 5 to 7 days of selection after each infection. Thus, singly transduced R cells were infected with p53^DD^ retroviruses (R+P) and conversely, singly transduced P cells were infected with Ras^G12V^ retroviral particles (P+R) (Figure 4A). The controls for each doubly transduced cell were prepared by using the empty vector used to clone the second transgene (pL or pB), establishing two populations termed P+pL and R+pB (Figure 4A). We also generated from primary adrenocortical cells a control cell population transduced with both empty vectors (pL+pB). Following appropriate antibiotic selection, the five resultant polyclonal cell populations were confirmed to express the desired transgenes with the exception of the pL+pB cells which did not express either one (Figure 4B). Importantly, the expression levels of the products of the introduced genes were comparable (Figure 4C).

The P+pL and R+pB control cell populations were indistinguishable from their singly infected P and R counterparts in terms of proliferative capacity and in their ability to form steroidogenic tissues once transplanted (Figure 4D; data not shown). Interestingly, the longer time in culture required for efficient selection did not alter the phenotype expressed by these cells, suggesting that no additional genetic changes had appeared during the process of generating these cells. The expression of constitutively active Ras followed by inactivation of wild-type p53 conferred to the cells a proliferation capacity similar to the PR cells (Figure 4D). On the contrary, the reverse order of gene transduction resulted in a marked reduction in the cell proliferation capacity (Figure 4D). To further characterize these cells, cell proliferation was determined by the percentage of Ki-67^+^ cells in each cell population. The R+P cell population maintained in 0.1% FCS-medium displayed a percentage of cells engaged in the cell cycle close to the percentage measured in PR cells, suggesting an almost complete independence from extrinsic mitogens for proliferation (Figure 4E). Conversely, the P+R cells exhibited a reduced proliferation index in the absence of mitogens and thus, a stronger dependence to growth factors (Figure 4E). Therefore, in cells with defective p53 signaling, oncogenic Ras, as a second genetic hit, is not able to totally substitute for a mitogenic signal. When the respective abilities of the R+P and P+R cells to form colonies in soft agar were compared, R+P cells were as efficient as the PR cells whereas the P+R cells were unable to grow, just like the R cells (Table 1). We then concluded from these *in vitro* experiments that R+P cells were transformed similarly to the PR cells.

**Figure 5:**
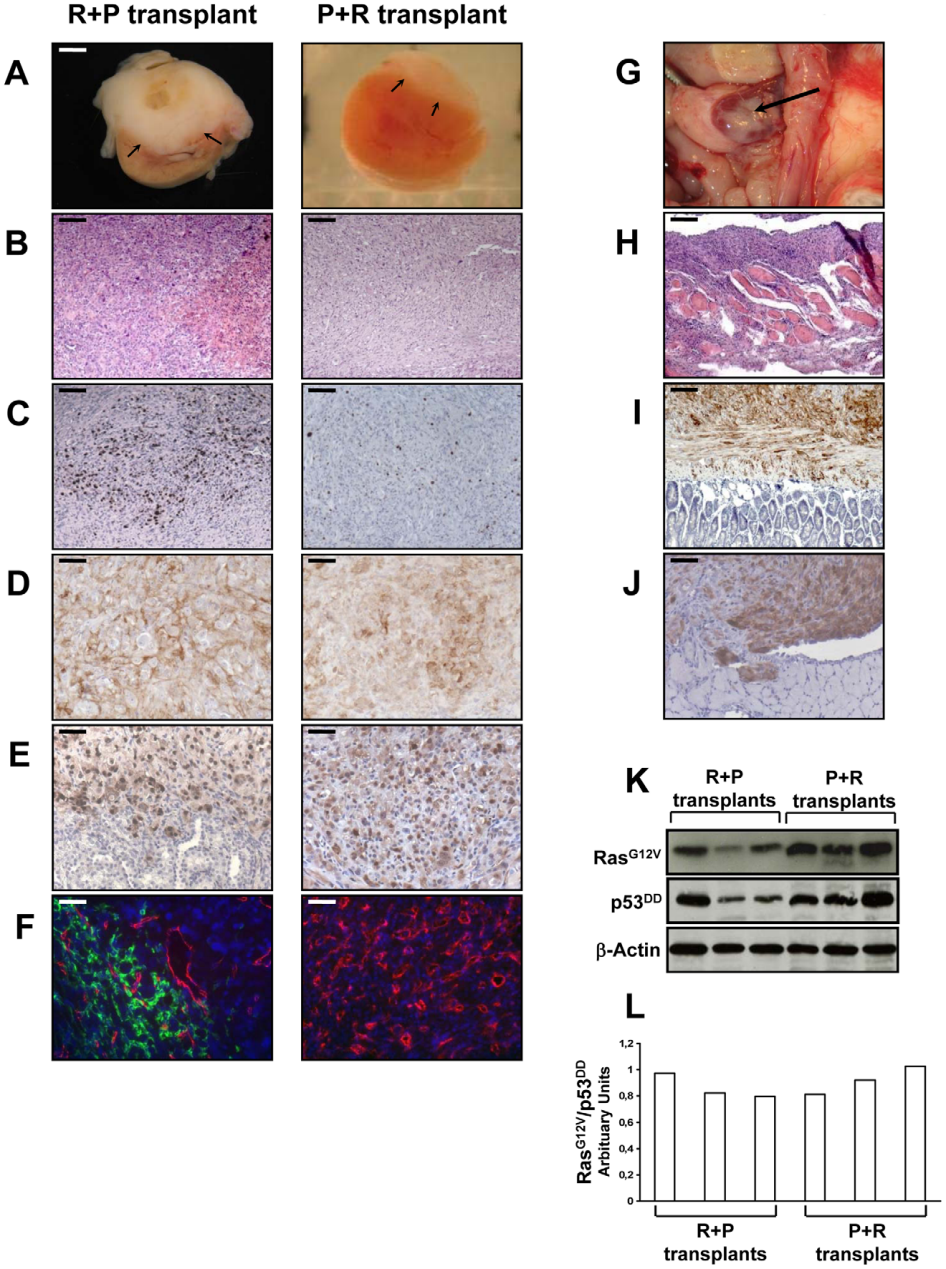
*In vivo* characterization of tissues formed at day 35 following the transplantation of R+P or P+R cells. A–F, 2×10^6^ R+P or P+R cells were transplanted under the kidney capsule of Scid mice. Xenografted tissue masses were removed from the animals at day 35 after cell transplantation and cut transversally showing internal tissue above the kidney (arrows) (A). Paraffin-embedded tissues were sectioned and H&E stained (bar, 100 µm) (B); assayed for Ki-67^+^ cells (bar, 100 µm) (C); immunostained for Ras (bar, 50 µm) (D); immunostained for p53 (bar, 50 µm) (E); and double CD31 (red) and LYVE-1 (green) immunofluorescence stained (no green fluorescence detected for the P+R transplant) (bar, 50 µm) (F). G–J, macroscopic intraperitoneal tumor spread on the spleen of mice after implantation of adrenocortical R+P cells (arrow) (G); and microscopic tumor spread in the abdominal muscle (H&E staining, bar, 400 µm) (H); in the intestines, immunostained for Ras (bar, 100 µm) (I); and in the diaphragm, immunostained for p53 (bar, 100 µm) (J); Western blot analysis of introduced genes. Expression of Ras^G12V^ and p53^DD^ was confirmed in three separate R+P and P+R tranplants. Actin served as a loading control (K). Ratio of Ras^G12V^ to p53^DD^ of R+P and P+R transplants (L).

The transplantation of R+P cells resulted in highly neoplastic (Figure 5A, 5B), proliferative (Figure 5C) and poorly differentiated tumors that invaded the kidney parenchyma and adjacent organs such as muscle, pancreas and adipose tissue (data not shown). At the time of necropsy, the primary tumors spread from the kidney to the spleen, abdominal muscle, intestinal mesentery and diaphragm (Figure 5G–5J). Therefore, R+P cells were as potent as PR cells to induce the formation of a metastatic adrenocortical carcinoma. In contrast, the tissues formed following the transplantation of P+R cells were benign tumors with no signs of kidney parenchyma invasion (Figure 5A, 5B) or metastases. The number of Ki-67^+^ cells was lower than in the R+P tumors (Figure 5C). This milder phenotype in P+R tissues could not be due to a difference in the level of expression of both transgenes as the levels of expression of p53^DD^ and Ras^G12V^ detected by immunohistochemistry were very similar in PR, R+P and P+R (Figure 5D, 5E). When the respective ability of R+P and P+R tissues to develop lymphatics was compared, a close correlation with the metastatic potential was found (Figure 5F) since P+R tumors were devoid of lymphatic vessels. We next sought to confirm the immunostaining and performed Western analyses of the levels of expression of p53^DD^ and Ras^G12V^ proteins in three R+P and P+R transplants. We noticed that the Ras^G12V^ and p53^DD^ protein levels appeared slightly lower in the R+P transplants than in the P+R transplants (Figure 5K). The malignant tumors generated by the transplantation of R+P cells is always more heterogenous than is benign counterparts, this is partly due to the invasion of the mouse adjacent tissues (kidney, pancreas and muscle) and also by the recruitment of other mouse cells such as stromal cells and fibroblasts. To confirm this, we examined the Ras^G12V^ to p53^DD^ ratio and found that the results between both groups yielded similar values (Figure 5L). These results further support the notion that, in this model system, the phenotype difference is mainly due to the acquisition order of the genetic alterations rather than the absolute expression level of the transgenes.

The tumor suppression function of p53 relies on its ability to act as a potent sequence-specific transcriptional activator, regulating a program of gene expression. In particular, p53 transactivates expression of the CDK inhibitor p21^WAF1/Cip1^. Therefore the lack or decrease of p21 expression will be a marker of the p53 function loss. As shown on Figure S2, pL and R cells expressing p53 demonstrated a strong p21 expression in almost all nuclei. In contrast, inactivation of p53 resulted in an absence or a marked decrease of p21 expression in P, P+R, R+P tissues and in an intestinal metastasis (Figure S2).

### Establishing a set of genes differentially expressed in benign versus malignant adrenocortical cells

**Figure 6:**
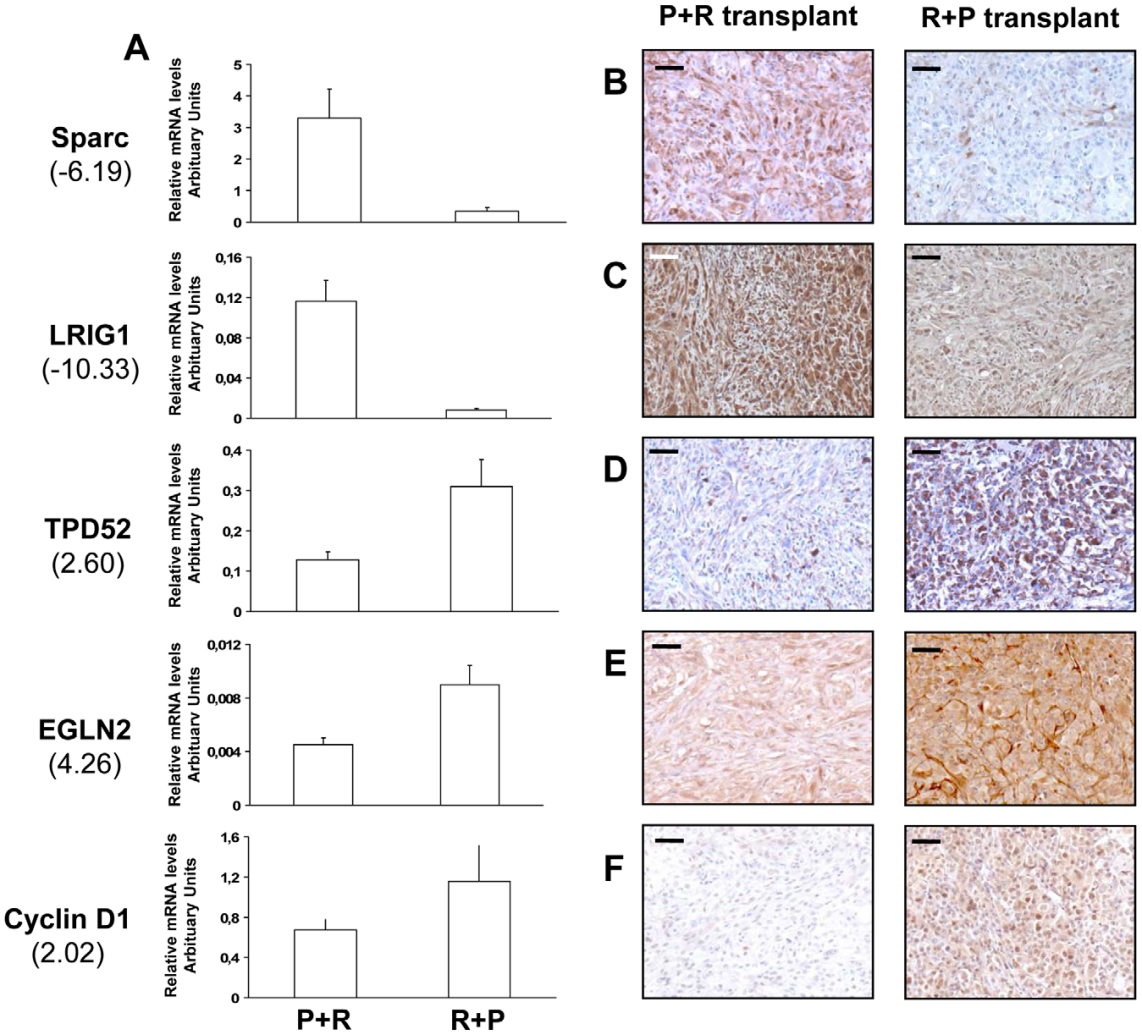
mRNA levels of *Sparc*, *LRIG1*, *TPD52*, *EGLN2*, and *CCND1* as measured by QRT–PCR in the P+R and R+P cells and proteins levels of these differentially expressed genes as measured by immunohistochemistry in tissues formed from transplanted P+R and R+P cells. Between parentheses, is indicated the fold change of expression levels of these genes as determined by microarray analysis between P+R and R+P cell populations (see Table S1). Relative expression levels normalized to RP-L27 were determined using specific gene-specific primers in the indicated cell population (A). Immunohistochemical protein staining for Sparc (B); LRIG1 (C); TPD52 (D); EglN2 (E); and Cyclin D1 (F) in P+R transplant and in R+P transplant (bars, 100 µm).

To identify patterns of gene expression associated with fully malignant behavior, we performed a transcriptomic microarray analysis on two P+R and three R+P cell populations. The gene list obtained from a class comparison between benign and malignant populations was filtered to ensure a 1.3 fold or higher change in expression level between the two groups. A total of 468 genes (499 probe sets) met this criterion. Among them, 157 were involved in cancer development and progression, 40 were over-expressed and 117 were under-expressed in the R+P cell populations compared to the P+R cell populations (Table S1). The other genes are involved in diverse cellular functions, including cell-to-cell signaling and interaction, transcription, cell death and metabolism. These results indicated that the distinct orders of genetic hits acquisition leads to distinct transcriptome signatures associated with a very distinctive *in vivo* phenotype. We further did real-time RT-PCR analysis of 5 genes known to be involved in tumor development and progression: *secreted protein*, *acidic*, *cysteine-rich* (*osteonectin*, *Sparc*), *leucine-rich repeats and immunoglobulin-like domains 1* (*LRIG1*), *tumor protein D52* (*TPD52*), *egl nine homolog 2* (*EglN2*) and *cyclin D1* (*CCND1*) using RNA from cells used in the microarray analysis. The expected differential expression was confirmed in all genes tested. *Sparc* and *LRIG1* were under-expressed in R+P cells compared with P+R cells (Figure 6A). *EglN2*, *TPD52* and *CCND1* were over-expressed (Figure 6A).

To validate these malignant to benign tumor transcriptome changes we performed immunohistochemical analysis on tissues formed after cell transplantation on the 5 genes already validated by QRT-PCR. For the 5 genes tested we confirmed the mRNA differential expression at the protein level: Sparc, LRIG1 proteins were under-expressed while TPD52, EglN2 and cyclin D1 proteins were over-expressed in the R+P tumors compared to P+R tumors (Figure 6B–6F).

## Discussion

Our present study establishes a new experimental *in vivo* system for understanding the pathogenesis of ACT. Starting with primary BAC cells, we have successfully transformed such cells into either benign or highly malignant and metastatic tumor-forming cells through the perturbation of one or two signaling pathways, respectively. Evidence has revealed that expression of both H-Ras^G12V^ and LT antigen was sufficient to convert primary BAC cells into fully malignant tumor cells [22]. However, the requirement of LT for transformation renders the analysis of these results complicated since LT viral oncoprotein is known to have several functions and to target a wide range of cellular proteins [26]. Moreover, the SV40 proteins are rarely involved in the etiology of human cancers [27]. However, ablation of both mammalian pRB and p53 tumor suppressor pathways has been recently shown to be sufficient to replace the function of LT oncoprotein in the combination of genes to transform normal human cells [28].

The significant progress made in identifying genetic alterations involved in ACT has been used as a platform to discriminate those that we believe might reasonably be involved in multistage tumorigenesis [11], [12]. The proto-oncogene *N-Ras* has been found to be mutated in 12.5% of ACA and ACC [13]. The involvement of this alteration in ACT development might appear relatively weak; however, it is noteworthy that overexpression of the epidermal growth factor receptor (EGFR/c-erbB1) is present in 3 to 43% of ACA and 76 to 100% of ACC [29]–[31], and is frequently associated with an overexpression of TGF-α, a natural ligand of EGFR in ACC [30]. As the signal transduced by EGFR tyrosine kinase activity involves Ras proteins among others, it is conceivable that Ras is activated in a larger proportion of ACT. Chronically active wild type Ras might promote tumorigenesis through activation of multiple Ras effectors that contribute to deregulated cell growth, dedifferentiation, and increased survival, migration and invasion. Somatic mutations in the *TP53* tumor suppressor gene occur in 25% to 33% of ACC but not in benign tumors [32], [33], suggesting that mutations in *TP53* participate in tumor progression rather than in initiation. Moreover, *TP53* inactivating mutations have been shown to identify a sub-group of ACC patients developing an aggressive tumor associated with a poor outcome [34]. Patients with *TP53* mutation also showed a trend towards a shorter survival duration [32], [33].

Several models of human or swine cell transformation using distinct combinations of mammalian genetic elements have been published in recent years [35]–[38]. Each model addressed the tumorigenic conversion of mammalian cells using various sequences of transgene introduction into target cells. Among these studies, the number of genetic events necessary for full cell transformation appeared to vary from 4 to 6. It is worth reminding that implantation of transformed cells in the subcutaneous space, as used in these studies, does not allow the survival of cells unless they are fully tumorigenic and, as a consequence, is not adapted for the study of the premalignant stages. In contrast, the SRC site is an advantageous niche for survival and growth of cells, due probably to the immediate access to oxygen and nutrients and to the rapid angiogenic response developed from the dense developed renal vasculature. Indeed, our previous studies on adrenocortical cells showed that injection of normal primary BAC cells under the kidney capsule was an important feature for the successful reconstruction of a functional and vascularized tissue, whereas these same cells did not survive when placed subcutaneously [39]. Moreover, although the kidney represents an ectopic site for adrenocortical cells, the tissues formed beneath the SRC recapitulate histological features characteristic of normal or pathological adrenal cortex [39]–[41].

The ability to form functional tissues from three independent R polyclonal populations contrasted with the report showing that high levels of ectopically activated Ras protein may result in premature senescence [42]. Therefore, there may exist a selection against cells overexpressing Ras^G12V^, leaving a population with moderate expression of the activated oncogene. The level of Ras expression would be then sufficient to activate one or more downstream signaling pathways controlled by Ras, such as the MAP kinase or the PI3 kinase pathways to a level that is essential to disrupt the fine balance between differentiation and proliferation, and to trigger some irreversible changes towards a benign phenotype.

To our knowledge, no other study has derived cell cultures from the same batch of initial cells and, after transduction with defined genetic elements in different orders, evaluated their effects on the expressed phenotype in an *in vivo* experimental model. In this tissue reconstruction model, each singly infected cell population produced a distinctive phenotype which might be thoroughly examined. Significantly, these findings show that malignant progression in ACT might be controlled not only by the acquisition of specific genetic changes but also, and more importantly, with their order of acquisition as we found that only BAC cells expressing Ras and p53^DD^ -in that order- could form carcinomas. This supports the prediction that overexpression or mutation in Ras signaling pathway mediate important early events underlying later tumorigenesis. Sun et al. noted that the order of introduction of Ras^G12V^ and LT did not affect the outcome of the transplantation; both cell populations formed very aggressive tumors [24]. One possible explanation is that, LT being such a powerful viral oncoprotein, the requirement for cooperation with Ras^G12V^ might be minimal whatever the order of acquisition is. Currently, we do not know how the order of acquisition of genetic alterations impacts the underlying mechanism of cooperation leading to different tumor phenotypes but this is the focus of our in progress investigations. Mouse models have been developed to dissect the interplay between mutant p53 and oncogenic Ras in human cancer and have demonstrated that the presence of both genetic alterations give rise to highly invasive and metastatic tumors associated with a decrease in survival [43]–[45]. According to our results, a model of pancreatic tumor progression involving initiation through K-Ras oncogenic mutation and progression through acquisition of p53 point mutation has been suggested, however the reverse combination was not studied [43]. Two p53 target genes, *BTG2* and *ATF3*, have been identified as mediators of the ability of wild-type p53 to resist Ras oncogenic transformation through reduced growth rate, anchorage independent growth and tumor formation in mice [46], [47]. The establishment of malignant phenotype for a transformed cell resides in the acquisition of new biological properties such as cellular motility, which makes possible invasion and metastasis. RhoA, a small GTPase involved in the cell motility process has been found to be negatively regulated by functional p53 and positively regulated by H-Ras^V12^; both signals resulting in a basal level of activated RhoA Upon loss of p53 function, RhoA activation increases, which in turn induces cell motility and disease progression [48]. Recently, microarray analysis of immortalized human fibroblasts transformed by the expression of H-Ras^G12V^ and inactivation of p53 identified a NFκB-dependent pro-inflammatory gene signature endowing these cells with an increased tumorigenicity [49], [50].

In our experimental model of adrenocortical tumorigenesis, BAC cells from the same genetic background acquire two alterations that in turn deregulate major cellular signaling pathways. If the order of the introduced transforming genes was irrelevant to the phenotype, then the tumor formed following transplantation should be identical. This was not the case, however. A transcriptomic analysis using cDNA microarrays has been used to identify the molecular signature that might explain the distinctive *in vivo* phenotypes. The analysis of P+R and R+P cell populations identified 468 differential genes and among those 157 genes directly involved in cancer development and progression that were differentially expressed between partially and fully transformed cells. Moreover, histochemical validation done on a subset of 5 gene products further confirmed their differential expression in malignant versus benign tumors formed after transplantation of these cells. The 5 genes were chosen on their apparent importance in tumor development and cancer progression. Sparc is an extracellular matrix-associated glycoprotein and a lower Sparc expression is correlated with increased growth, metastatic behavior and reduced apoptosis in multiple cancers [51]. LRIG1 is a transmembrane protein acting as a negative feedback regulator of EGF signaling [52]. Its expression is downregulated in a variety of human cancer supporting the hypothesis that decreased expression of LRIG1 unleashes EGFR signaling, which might contribute to tumorigenesis. To date no data are available on LRIG1 status in ACC where EGFR expression is markedly elevated [29]–[31]. However, LRIG1 expression has also been shown to be up-regulated in prostate cancer and leukemia which highlighted that LRIG1 might act as an oncogene depending on the cellular contexts [53]. Increased TPD52 expression and gene copy number have been reported in breast, prostate and ovarian cancer increasing cell proliferation *in vitro* and tumorigenicity in mice [54]–[57]. Cyclin D1 overexpression driven by genomic alterations, post-transcriptional regulation, or post-translational protein stabilization is implicated as driving feature in various human tumors [58]. Finally, level of EglN2, a prolyl hydroxylase, has been shown to be significantly higher in human renal clear cell carcinoma than in normal kidneys [59]. Moreover, inactivation of EglN2 down-regulated Cyclin D1 and cell proliferation in several cancer cell lines [60]. Increased EglN2 expression in R+P cells compared to P+R cells might participate to higher cell proliferation through Cyclin D1 regulation.

Thus far, we have employed a rational modeling approach to improve our understanding of the genetic changes leading to the initiation and progression of adrenocortical cancer and to shed some light on the critical importance of the order of genetic alterations for the tumor development. We have focused on Ras and p53 genes because modifications in their expression and/or in their genomic sequence are commonly observed in human ACT. Other genes such as *IGF-2*, *β-catenin*, *H19*, *p57*, *EGFR* have also been shown to play a role in adrenal pathogenesis and need to be tested in future studies. Hence, the system that we established will enable us to test the oncogenic potential of these genes singly or in combination, in order to identify those that might truly contribute to adrenocortical tumor development and those that might only be bystanders. We are confident that other gene combinations will lead to ACT development with some specific clinical and histopathological features and it will be then possible to link the genotypes with the tumor phenotype. Finally, the first identification of the minimal combination of two master pathways sufficient to trigger ACC development will help to design new therapeutic options targeting these specific gene products or the downstream targets of their signaling pathways.

## Materials and Methods

### Ethics statement

Animal use was conducted according to the institutional guidelines and those formulated by the European Community for the Use of Experimental Animals. The animal protocol was approved by the Institutional Animal Care and Use Committee at the Commisariat à l'Energie Atomique.

### Plasmid construction and production of retroviral particles

The *H-Ras^G12V^* cDNA previously inserted into pBabe-Hygro (a gift from Pr. J.W. Shay; [61]), was subcloned into MoMLV derived vector pL (Clontech), downstream of the immediate early cytomegalovirus promoter. A dominant negative p53 fragment, *p53^DD^* cloned into pBabe-Hygro was purchased from Addgene (plasmid 9058) [14]. pL-Ras^G12V^ (resistant to neomycin) and pBabe-Hygro-p53^DD^ (resistant to hygromycin) constructs and the corresponding empty retroviral vectors were used to transfect the amphotropic packaging cell line PT67 (Clontech) using the Effecten Transfection Reagent (Life Technologies Invitrogen). The cells underwent selection with 400 µg/ml neomycin for 10 days or 50 µg/ml hygromycin for 6 days. Then, the viral supernatant was collected and filtered through a 0.45 µm syringe filter to obtain cell-free viruses for adrenocortical cell infection.

### Culture of BAC cells and retroviral transduction

Primary adrenocortical cells were prepared by dissection and enzymatic digestion of adrenal glands from 2-yr-old steers [62]. They were grown at 37°C under a 5% CO_2_-95% air atmosphere in DMEM/Ham's F-12 1∶1 supplemented with 10% FCS, 10% horse serum and 1% (v/v) UltroSer G (BioSepra) (complete medium). When reaching 40–50% confluence, BAC cells were infected by a mix of two retroviral suspensions for 24 hours (pBabe-Hygro-p53^DD^/pL-Ras^G12V^ or pBabe-Hygro-p53^DD^/pL or pBabe-Hygro/pL-Ras^G12V^). Infected cells were selected with 400 µg/ml G418 for 7 days and 50 µg/ml hygromycin for 5 days to obtain stable cell lines.

In different experiments, primary cells were transduced with a single retrovirus, pL-Ras^G12V^ or pBabe-Hygro-p53^DD^ and selected with G418 for 7 days or hygromycin for 5 days, respectively. Stably infected cultures were then infected with either pBabe-Hygro or pBabe-Hygro-p53^DD^, or pL or pL-Ras^G12V^, respectively, and selected with G418 for 7 days and hygromycin for 5 days to obtain stable cell lines. Cells were not grown extensively between the two infections. Primary BAC cells transduced only with the empty vectors pL were used as control cells for the effect of the genes of interest. Three separate adrenocortical primary cell preparations have been used to generate the stably infected cells described above.

### In vitro analysis

Gene expression analysis was assessed by RT-PCR. One microgram of total RNA of cultured cells, prepared using the RNAgents Total RNA Isolation System (Promega), were reverse transcribed using the ImProm-II Reverse Transcriptase (Promega) with random primers PdN6 (Life Technologies Invitrogen); after which 2 µL of each reaction were PCR amplified using following primers: 5-ATGACGGAATATAAGCTGGTGGT and 5-TCAGGAGAGCACACACTTGC (Ras^G12V^), 5-AAAGGATGCCCATGCTACAG and 5- TTGCCGGGAAGCTAGAGTAA (p53^DD^), and 5-GCGGCTATCGTGAAGAACATTG and 5-CCTTGCGTTTGAGAGCAGGG (RP-L27; Ribosomal Protein-L27).

Proliferation was determined by assessing cell number after growth in complete medium, for 7 days (5×10^3^ cells of each cell line were initially plated). Each day, cells were counted in triplicate using a Coulter Z1 (Coultronics). Proliferation was also assessed by the percentage of Ki-67 positive cells in each cell population. For each cell line, 10^4^ cells were plated in complete medium for 24 hours in 4 well LabTek chamber slide (Fisher Scientific). After deprivation in DMEM/0.1% FCS for 48 hours, cells from two wells were transferred in complete medium whereas cells from the two other wells were maintained in 0.1% FCS-medium for 24 hours. Cells were then fixed in 4% paraformaldehyde and immunostained with the anti Ki-67 monoclonal antibody (clone MIB-1; Dako). An average of 300 nuclei were counted on each well, *n* = 4 for each experimental condition.

5×10^3^ cells of individual cell lines were seeded in triplicate in soft agar and the resulting colonies were scored three weeks later. Each experiment was repeated at least once.

### Cell transplantation and animal experimentation

Both male and female SCID mice, originally purchased from Taconic, were used at an age greater than 6 weeks (∼25 g body weight) in these experiments. Under tribromoethanol anesthesia, mice were adrenalectomized and 2×10^6^ genetically modified adrenocortical cells were transplanted under the kidney capsule [39], [63]. Six mice were used per polyclonal cell population generated. Post-operative care for the animals consisted of the administration of analgesics and antibiotics in drinking water for 4 days [39]. Animals were killed at various times, from 8 to 35 days after transplantation and subjected to necropsy.

### Histological and immunohistochemical analysis

All tissues (adrenocortical transplants and metastases) were fixed in 4% paraformaldehyde and embedded in paraffin. Microtome sections (5 µm thick) were stained with H&E for histological analysis. Expression of the transduced genes was analyzed by standard immunohistochemistry using the anti-Ras mouse monoclonal antibody (clone 18; BD Transduction Laboratories), the anti-p53 mouse monoclonal antibody (clone pAb421; Calbiochem), and the anti-p21 mouse monoclonal antibody (clone EA10; Calbiochem) detected with a biotin-conjugated anti-mouse IgG antibody and an avidin-biotin-peroxidase complex (Vector Laboratories). Sections were counterstained with hematoxylin. The differentiation status and proliferation index of tissues were determined using a rabbit polyclonal anti-3βHSD antibody (produced in our laboratory) and the MIB-1 antibody that recognizes the proliferation-associated Ki-67 antigen, respectively. The number of Ki-67 positive cells per 100 BAC cells was designated as the proliferation index. Counting was performed using two non-consecutive tissue sections per tissue sample, selected at random in each group. DNA fragmentation associated with apoptosis was detected by nick end labeling of sections using the TdT-FragEL kit (TUNEL) (Calbiochem). Vascular endothelial cells were labeled with a rat monoclonal anti-CD31 antibody (PECAM-1; BD Biosciences) and lymphatic endothelial cells were labeled with a goat polyclonal anti-LYVE 1 antibody (R&D Systems), on paraffin-embedded sections (5 µm thick) of tissues fixed in Accustain formalin free fixative (Sigma-Aldrich). Secondary antibodies were Cy3- or FITC-labeled donkey anti-rat IgG or anti-goat IgG respectively. Sections were counterstained with DAPI.

### Western blot analysis

Cells were rinsed in PBS and lysed in RIPA buffer (10 mM Tris-HCl, pH 7.4, 150 mM NaCl, 1% TritonX-100, 0.5% Na deoxycholate, 0.1% SDS) supplemented with protease inhibitor cocktail (#P8340; Sigma) for 10 minutes on ice, scrapped from the culture dish, and cleared with centrifugation in a microfuge tube for 20 min at 4°C. Extracts were analyzed for protein concentration by Bradford assay. Equal amount (25 µg) of total cell protein was separated by 15% SDS-PAGE gel and transferred to nitrocellulose membrane (Bio-Rad Laboratories). Filters were blocked for 1 hr at room temperature in 5% dry milk in Tris Buffered Saline, and incubated with primary antibodies in 5% dry milk in TBS at 4°C overnight. The following primary antibodies were used: anti-Ras mouse monoclonal antibody (clone 18; BD Transduction Laboratories), anti-p53 mouse monoclonal antibody (clone pAb421; Calbiochem), anti-actin mouse monoclonal antibody (clone AC-15; Sigma-Aldrich). After several washes, secondary peroxidase conjugated antibodies (Thermo Scientific) were used at a 1∶10000 dilution. The membrane was washed in TBS-5% Tween 20 and the proteins were detected using an enhanced chemiluminescence (ECL) detection system (Amersham).

For protein isolation from tissues, the xenograft is carefully dissected out from the kidney and the adjacent tissues when possible and finely cut with a razor blade into a mortar with approximately 500 µl of RIPA lysis buffer containing a protease inhibitor cocktail. The tissue was ground well with a pestle, transferred into a microfuge tube and centrifuged for 20 min at 4°C. Protein extracts were then submitted to the same protocol as described above. Quantification was done with Image J image software (National Institute of Health).

### Microarray analysis

#### RNA isolation and target labeling

Total RNA was extracted from two P+R, two pBabe+pL (control for P+R cells), three R+P and two pL+pBabe (control for R+P cells) cell populations using RNeasy kit (Qiagen).

Microarray analysis was performed by the ProfileXpert platform (Lyon, France) using a high-density oligonucleotide array (GeneChip Bovine Genome array, Affymetrix). One microgram of total RNA from each sample was amplified and biotin-labeled using GeneChip Expression 3′ Amplification One-Cycle Target Labeling and Control Reagents Kit. Before amplification, spikes of synthetic mRNA at different concentrations were added to all samples; these positive controls were used to ascertain the quality of the process. Biotinylated antisense cRNA for microarray hybridization was prepared. After final cleanup, cRNA quantification was performed with a NanoDrop and quality checked with Agilent 2100 Bioanalyzer (Agilent Technologies, Inc).

#### Arrays hybridization and scanning

Hybridization was performed following Affymetrix protocol (http://www.affymetrix.com). Briefly, 10 µg of labeled cRNA was fragmented and denaturated in hybridization buffer, then hybridized on chip during 16 hours at 45°C with constant mixing by rotation at 60 rpm in an Genechip hybridization oven 640 (Affymetrix). After hybridization, arrays were washed and stained with streptavidin-phycoerythrin (Invitrogen) in a fluidic 450 (Affymetrix) according to the manufacturer's instruction. The arrays were read with a confocal laser (Genechip scanner 3000, Affymetrix). Then CEL files were generated using the Affymetrix GeneChip Command Console (AGCC) software 3.0.

#### Data filtering and analysis

The obtained data were normalized with Affymetrix Expression Console software using MAS5 statistical algorithm. Normalized data were compared and filtered using Partek Genomic Suite software 6.5 (Partek Inc.). Pair-wise comparisons were performed between carcinoma samples and control samples. Each sample from one group was compared with each sample from the other group and only genes showing a variation of 1.3-fold in all pairwise comparisons were retained. Then, a gene was considered as differentially expressed between groups only if the detected signal was above the background for at least one of the compared groups. The same comparison was performed between adenomas and control samples. Finally, genes differentially expressed in carcinomas and adenomas were compared to each other and only genes showing a variation of 1.3-fold in all pairwise comparisons were retained. The genes of interest were then listed and classified according to their biological functions using Ingenuity Pathways Analysis (Ingenuity Systems Inc.).

All data is available at the GEO public data base under accession GSE32305.

### Quantitative real-time PCR

One microgram of total RNA prepared for the microarray hybridisation was used to generate cDNAs by reverse transcription using the iScript system (Bio-Rad) as recommended by the manufacturer. Real-time PCR was performed using Bio-rad CFX96 apparatus and qPCR Master Mix (Promega). The values for the specific genes were normalized to the *RP-L27*. Specific primers sequences are provided in Table S2.

## Supporting Information

Figure S1 Tumor growth analysis at day 8, 14 and 21 after transplantation of PR cells. After growth in culture, PR cells were transplanted beneath the kidney capsule of Scid mice. A, macroscopic appearance of kidney and xenografted tissue mass removed from the animals at day 8, 14, 21 after transplantation of 2×10^6^ PR cells. Adrenocortical tissue and kidney were cut transversally showing tissue expansion over time (arrows). B–E, paraffin-embedded tissues were sectioned and stained with H&E (bar, 400 µm) (B); assayed for Ki-67^+^ cells (bar, 100 µm) (C); or immunostained for Ras revealing invasion into adjacent muscle tissue at day 21 (bar, 50 µm) (D); or immunostained for p53 revealing invasion into adjacent adipose tissue at day 21 (bar, 50 µm) (E).
(PPT)

Figure S2 Qualitative changes of p21 expression in tissues formed after transplantation of pL, R, P, P+R, R+P cells and in intestinal metastasis issued from a R+P primary tumor. Paraffin-embedded tissues were sectioned and immunostained for p21 (bar, 50 µm). The junction with the mouse kidney is visible at the bottom of the tissue pictures. Intestinal villi are visible on the left of the metastasis picture. Arrows indicate stained nuclei.
(PPT)

Table S1 Differential expression of 157 genes related to cancer development and progression resulting from microarray analysis of R+P and P+R cell populations
(DOC)

Table S2 Primers for QRT–PCR.
(DOC)
