# Supplementary figures and images for: Acquisition Order of Ras and p53 Gene Alterations Defines Distinct Adrenocortical Tumor Phenotypes
Source: PLoS Genet. 2012 May 10;8(5):e1002700. doi: 10.1371/journal.pgen.1002700 (PMC3349738; doi:10.1371/journal.pgen.1002700)

## Slide 1
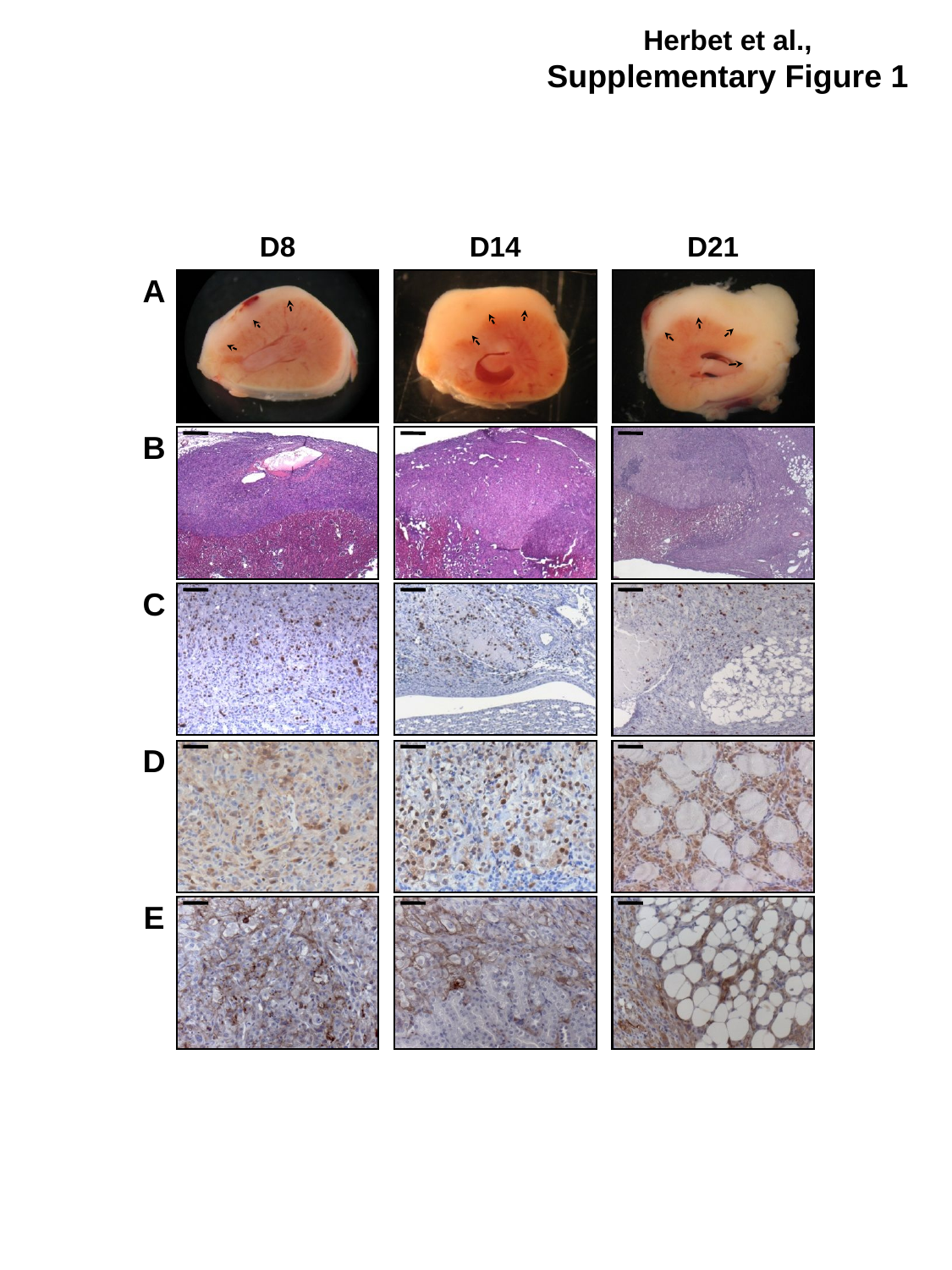

Herbet et al.,
Supplementary Figure 1
D8
D14
D21
A
B
C
D
E

Supplement: Figure S1 — Tumor growth analysis at day 8, 14 and 21 after transplantation of PR cells. After growth in culture, PR cells were transplanted beneath the kidney capsule of Scid mice. A, macroscopic appearance of kidney and xenografted tissue mass removed from the animals at day 8, 14, 21 after transplantation of 2×10^6^ PR cells. Adrenocortical tissue and kidney were cut transversally showing tissue expansion over time (arrows). B–E, paraffin-embedded tissues were sectioned and stained with H&E (bar, 400 µm) (B); assayed for Ki-67+ cells (bar, 100 µm) (C); or immunostained for Ras revealing invasion into adjacent muscle tissue at day 21 (bar, 50 µm) (D); or immunostained for p53 revealing invasion into adjacent adipose tissue at day 21 (bar, 50 µm) (E). (PPT) [file pgen.1002700.s001.ppt]

## Slide 1
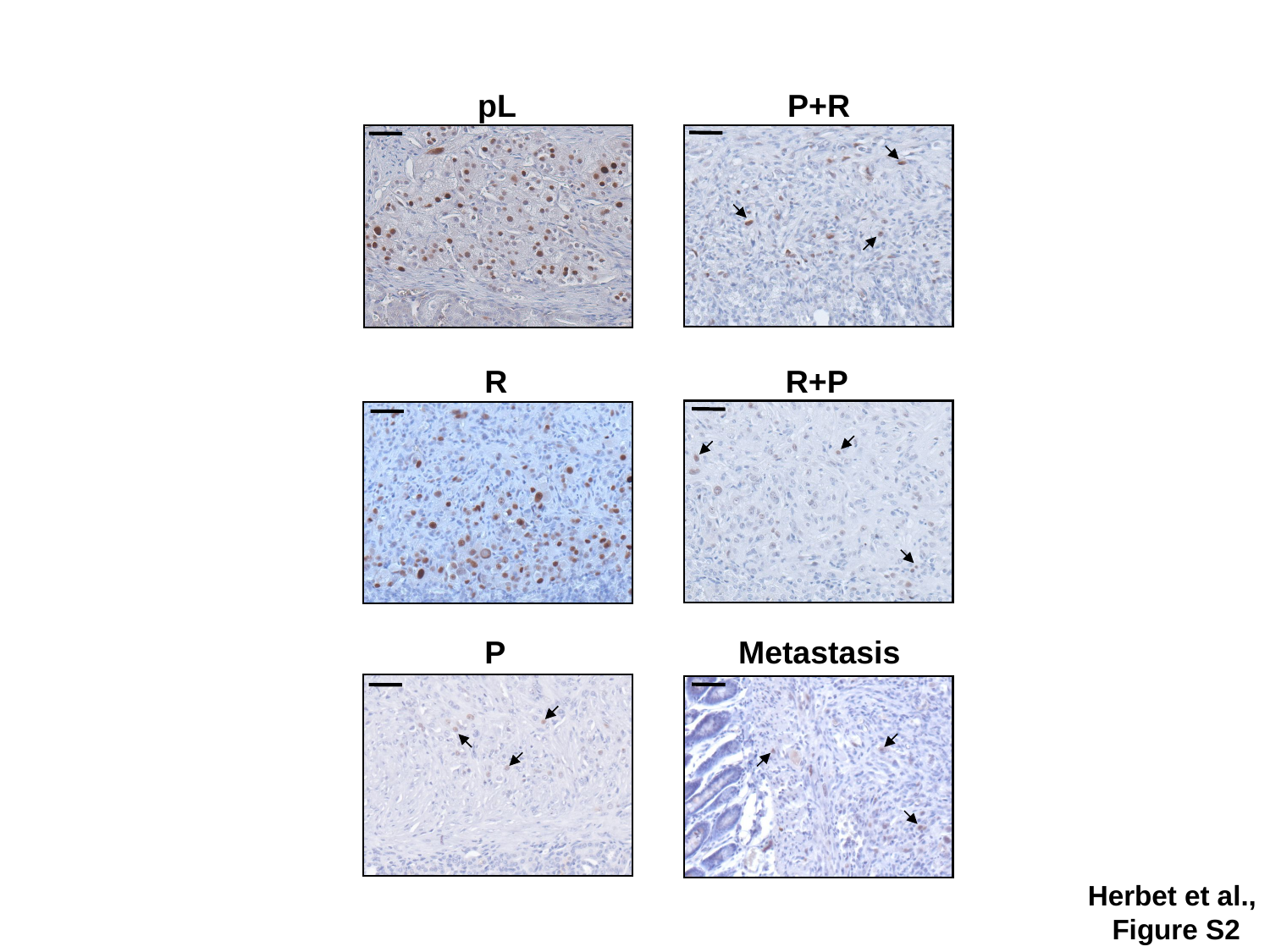

pL
P+R
R
R+P
P
Metastasis
Herbet et al.,
 Figure S2

Supplement: Figure S2 — Qualitative changes of p21 expression in tissues formed after transplantation of pL, R, P, P+R, R+P cells and in intestinal metastasis issued from a R+P primary tumor. Paraffin-embedded tissues were sectioned and immunostained for p21 (bar, 50 µm). The junction with the mouse kidney is visible at the bottom of the tissue pictures. Intestinal villi are visible on the left of the metastasis picture. Arrows indicate stained nuclei. (PPT) [file pgen.1002700.s002.ppt]
