# Supplementary material for: Acquisition Order of Ras and p53 Gene Alterations Defines Distinct Adrenocortical Tumor Phenotypes
Source: PLoS Genet. 2012 May 10;8(5):e1002700. doi: 10.1371/journal.pgen.1002700 (PMC3349738; doi:10.1371/journal.pgen.1002700)
Supplement: Table S1 — Differential expression of 157 genes related to cancer development and progression resulting from microarray analysis of R+P and P+R cell populations (DOC) [file pgen.1002700.s003.doc]

**Supplementary Table 1**: Differential expression of 157 genes related to cancer development and progression resulting from microarray analysis of R+P and P+R cell populations.

1. Overexpressed genes

| **Probe Set ID** | **Gene Symbol** | **Gene Title** | **RefSeq Transcript ID** | **Fold Change** | ***P* value** |
| --- | --- | --- | --- | --- | --- |
| Bt.1666.1.A1_at | TSG101 | tumor susceptibility gene 101 | NM_001097995 | 1,43634 | <0.05 |
| Bt.4535.1.S1_at | YARS | tyrosyl-tRNA synthetase | NM_174220 | 1,55271 | <0.05 |
| Bt.22141.1.S1_at | JUP | Junction plakoglobin | NM_001004024 | 1,77659 | <0.05 |
| Bt.27793.1.A1_at | MLLT3 | myeloid/lymphoid or mixed-lineage leukemia (trithorax homolog, Drosophila); translocated to, 3 | XM_600235 | 1,78098 | <0.05 |
| Bt.20548.1.S1_at | N4BP1 | similar to Nedd4 binding protein 1 | XR_042901 | 1,8388 | <0.05 |
| Bt.22737.2.S1_at | ERBB2IP | erbb2 interacting protein | XM_865008 | 1,85555 | <0.05 |
| Bt.4539.1.S1_at | AGTR1 | angiotensin II receptor, type 1 | NM_174233 | 2,00715 | <0.05 |
| Bt.12562.1.S1_at | CAPN5 | calpain 5 | XM_617141 | 2,02321 | <0.05 |
| **Bt.24862.1.A1_at** | **CCND1** | **cyclin D1** | **NM_001046273** | **2,02366** | <0.05 |
| Bt.17885.1.A1_at | FOSL1 | FOS-like antigen 1 | XM_609882 | 2,38564 | <0.05 |
| Bt.4586.1.S1_at | GNG2 | guanine nucleotide binding protein (G protein), gamma 2 | NM_174072 | 2,50884 | <0.05 |
| **Bt.27864.1.S1_at** | **TPD52** | **tumor protein D52** | **NM_001076922** | **2,60765** | <0.05 |
| Bt.19093.1.S1_at | ALDH1A2 | Aldehyde dehydrogenase 1 family, member A2 | XM_615062 | 2,69179 | <0.05 |
| Bt.4757.1.S1_at | ARHGDIB | Rho GDP dissociation inhibitor (GDI) beta | NM_175797 | 2,92245 | <0.05 |
| Bt.12694.1.S1_at | TNFRSF21 | tumor necrosis factor receptor superfamily, member 21 | NM_001076911 | 3,1324 | <0.05 |
| Bt.4818.1.S1_at | BEX2 | brain expressed X-linked 2 | NM_001077087 | 3,28234 | <0.05 |
| Bt.21912.1.S2_at | EIF2C2 | eukaryotic translation initiation factor 2C, 2 | NM_205794 | 3,30429 | <0.05 |
| Bt.25297.1.A1_at | ISOC1 | Isochorismatase domain containing 1 | NM_001101213 | 3,40394 | <0.05 |
| Bt.13534.1.S1_at | PLA2G16 | phospholipase A2, group XVI | NM_001075280 | 3,48178 | <0.05 |
| Bt.800.1.S1_at | MAPK13 | mitogen-activated protein kinase 13 | NM_001014947 | 3,58597 | <0.05 |
| Bt.4902.1.S1_at | CTSZ | cathepsin Z | NM_001077835 | 3,72564 | <0.05 |
| Bt.23135.1.S1_at | TAGLN2 | transgelin 2 | NM_001013599 | 3,79393 | <0.05 |
| Bt.22275.1.A1_at | ISG20 | interferon stimulated exonuclease gene 20kDa | XM_583075 | 3,90637 | <0.05 |
| **Bt.15798.1.S1_at** | **EGLN2** | **egl nine homolog 2 (C. elegans)** | **NM_001102193** | **4,26755** | <0.05 |
| Bt.4897.1.S1_at | INHA | inhibin, alpha | NM_174094 | 4,48208 | <0.05 |
| Bt.20613.2.A1_at | BCAR3 | breast cancer anti-estrogen resistance 3 | NM_001024483 | 4,85009 | <0.05 |
| Bt.26104.1.A1_at | WASF1 | WAS protein family, member 1 | NM_001034017 | 5,06078 | <0.05 |
| Bt.20923.1.S1_at | EIF2AK2 | eukaryotic translation initiation factor 2-alpha kinase 2 | NM_178109 | 6,49805 | <0.05 |
| Bt.18631.1.A1_at | SPRY4 | Sprouty homolog 4 (Drosophila) | NM_001081512 | 6,60936 | <0.05 |
| Bt.15802.2.S1_at | KLF5 | Kruppel-like factor 5 (intestinal) | NM_001083727 | 6,68467 | <0.05 |
| Bt.24033.1.A1_at | DDX58 | DEAD (Asp-Glu-Ala-Asp) box polypeptide 58 | XM_580928 | 7,09902 | <0.05 |
| Bt.191.1.S1_at | IL1A | interleukin 1, alpha | NM_174092 | 7,81858 | <0.05 |
| Bt.21250.1.S1_a_at | TMEM176B | transmembrane protein 176B | NM_001099145 | 8,96488 | <0.05 |
| Bt.20409.1.S1_at | PARP9 | poly (ADP-ribose) polymerase family, member 9 | NM_001076828 | 10,2464 | <0.05 |
| Bt.4856.1.S1_at | IL1B | interleukin 1, beta | NM_174093 | 12,4585 | <0.05 |
| Bt.12283.1.A1_at | DIRAS3 | DIRAS family, GTP-binding RAS-like 3 | NM_001034215 | 16,8949 | <0.05 |
| Bt.16857.1.A1_at | IFIH1 | interferon induced with helicase C domain 1 | XM_615590 | 17,0799 | <0.05 |
| Bt.8436.1.S1_at | IFI6 | interferon, alpha-inducible protein 6 | NM_001075588 | 18,8289 | <0.05 |
| Bt.12304.1.S1_at | ISG15 | ISG15 ubiquitin-like modifier | NM_174366 | 75,8906 | <0.05 |
| Bt.20785.2.S1_at | IFI44 /// LOC781857 | interferon-induced protein 44 /// similar to Interferon-induced protein 44 (Anti | XM_872122 /// XR_027350 | 122,738 | <0.05 |

1. Underexpressed genes

| **Probe Set ID** | **Gene Symbol** | **Gene Title** | **RefSeq Transcript ID** | **Fold Change** | ***P* value** |
| --- | --- | --- | --- | --- | --- |
| Bt.10398.1.S1_at | PTX3 | pentraxin-related gene, rapidly induced by IL-1 beta | NM_001076259 | -158,012 | <0.05 |
| Bt.23840.1.S1_at | PCSK6 | Proprotein convertase subtilisin/kexin type 6 | XM_603014 | -66,9232 | <0.05 |
| Bt.7012.1.S1_at | BCAS1 | breast carcinoma amplified sequence 1 | NM_001077850 | -48,5141 | <0.05 |
| Bt.20173.1.A1_at | DPT | dermatopontin | NM_001045903 | -47,8586 | <0.05 |
| Bt.2638.1.S1_at | SERPINF1 | serpin peptidase inhibitor, clade F (alpha-2 antiplasmin, pigment epithelium derived factor), member 1 | NM_174140 | -31,2422 | <0.05 |
| Bt.8106.1.S1_at | NID1 | nidogen 1 | NM_001101155 | -30,2384 | <0.05 |
| Bt.25926.1.A1_at | PDE3B | phosphodiesterase 3B, cGMP-inhibited | XM_618666 | -23,6721 | <0.05 |
| Bt.5351.1.S1_a_at | BGN | biglycan | NM_178318 | -21,0806 | <0.05 |
| Bt.3797.1.S1_at | FMOD | fibromodulin | NM_174058 | -20,9608 | <0.05 |
| Bt.2988.1.S1_at | GNG11 | guanine nucleotide binding protein (G protein), gamma 11 | NM_001024523 | -20,6465 | <0.05 |
| Bt.8963.1.S1_at | LOXL1 | lysyl oxidase-like 1 | NM_174383 | -19,8816 | <0.05 |
| Bt.9633.2.S1_at | MEOX2 | mesenchyme homeobox 2 | NM_001098045 | -19,6246 | <0.05 |
| Bt.4565.1.S1_at | SNAI2 | snail homolog 2 (Drosophila) | NM_001034538 | -19,3063 | <0.05 |
| Bt.13556.1.S1_a_at | CFH | complement factor H | NM_001033936 | -17,7694 | <0.05 |
| Bt.5395.1.S1_a_at | VCAN | versican | NM_181035 | -17,3952 | <0.05 |
| Bt.3540.1.S1_at | SFRP4 | secreted frizzled-related protein 4 | NM_001075764 | -16,3573 | <0.05 |
| Bt.13542.1.S1_at | CFB | complement factor B | NM_001040526 | -15,7625 | <0.05 |
| Bt.28583.1.S1_s_at | COL1A1 | collagen, type I, alpha 1 | NM_001034039 | -15,2234 | <0.05 |
| Bt.11694.1.A1_at | PRRX2 | paired related homeobox 2 | XM_613967 | -15,1307 | <0.05 |
| Bt.8948.1.S1_at | LOX | lysyl oxidase | NM_173932 | -14,7515 | <0.05 |
| Bt.7984.1.S1_at | LAMA4 | Laminin, alpha 4 | XM_608123 | -14,562 | <0.05 |
| Bt.27689.1.A1_at | WISP2 | WNT1 inducible signaling pathway protein 2 | NM_001102176 | -13,8165 | <0.05 |
| Bt.13670.1.S2_at | ALPL | alkaline phosphatase, liver/bone/kidney | NM_176858 | -13,194 | <0.05 |
| Bt.3202.1.A1_at | PRELP | proline/arginine-rich end leucine-rich repeat protein | NM_174434 | -12,9834 | <0.05 |
| Bt.27379.1.A1_at | CXCR7 | Chemokine (C-X-C motif) receptor 7 | NM_001098381 | -12,7884 | <0.05 |
| Bt.2452.1.S1_at | LUM | lumican | NM_173934 | -10,337 | <0.05 |
| **Bt.3393.1.S1_at** | **LRIG1** | **leucine-rich repeats and immunoglobulin-like domains 1** | **XM_582085** | **-10,3353** | <0.05 |
| Bt.5220.2.S1_at | ENG | endoglin | NM_001076397 | -10,2572 | <0.05 |
| Bt.12991.1.S1_at | FGF10 | fibroblast growth factor 10 | XM_001249706 /// XM_592611 | -10,2083 | <0.05 |
| Bt.29877.1.S1_at | PDGFRA | platelet-derived growth factor receptor, alpha polypeptide | XM_590921 | -10,1138 | <0.05 |
| Bt.13681.2.S1_at | COL6A1 | collagen, type VI, alpha 1 | NM_001143865 /// XM_588755 | -9,97625 | <0.05 |
| Bt.12297.1.S1_at | LOXL4 | lysyl oxidase-like 4 | NM_174384 | -8,6261 | <0.05 |
| Bt.9958.1.S1_at | IGFBP6 | insulin-like growth factor binding protein 6 | NM_001040495 | -8,22778 | <0.05 |
| Bt.5229.1.S1_at | SERPINE1 | serpin peptidase inhibitor, clade E (nexin, plasminogen activator inhibitor type 1), member 1 | NM_174137 | -8,20389 | <0.05 |
| Bt.25312.1.A1_at | COLEC12 | collectin sub-family member 12 | NM_001101843 | -7,9804 | <0.05 |
| Bt.23597.1.S1_at | NUPR1 | nuclear protein 1 | NM_001114515 /// XM_867457 | -7,87622 | <0.05 |
| Bt.29890.1.S1_at | ADAM12 | ADAM metallopeptidase domain 12 | NM_001001156 | -7,07852 | <0.05 |
| Bt.5525.1.S1_at | ECM1 | extracellular matrix protein 1 | NM_001099706 | -7,013 | <0.05 |
| Bt.1770.2.A1_at | PXDN | peroxidasin homolog (Drosophila) | XM_593953 | -6,6323 | <0.05 |
| Bt.21589.1.S1_at | ANGPTL2 | angiopoietin-like 2 | NM_001109814 | -6,55932 | <0.05 |
| Bt.12916.1.S1_at | GPX3 | glutathione peroxidase 3 (plasma) | NM_174077 | -6,51478 | <0.05 |
| Bt.725.1.S1_at | PDGFRL | platelet-derived growth factor receptor-like | NM_001035301 | -6,21746 | <0.05 |
| Bt.12498.1.A1_at | COL6A3 | collagen, type VI, alpha 3 | XM_609132 | -6,20377 | <0.05 |
| **Bt.6768.1.S1_at** | **SPARC** | **secreted protein, acidic, cysteine-rich (osteonectin)** | **NM_174464** | **-6,19141** | <0.05 |
| Bt.22969.1.S1_a_at | SERPING1 | serpin peptidase inhibitor, clade G (C1 inhibitor), member 1 | NM_174821 | -6,0421 | <0.05 |
| Bt.4865.1.S1_at | EMILIN1 | Elastin microfibril interfacer 1 | XM_594120 | -5,8351 | <0.05 |
| Bt.5011.1.S2_at | LTBP2 | latent transforming growth factor beta binding protein 2 | NM_174385 | -5,8047 | <0.05 |
| Bt.5522.1.S1_at | THBS2 | thrombospondin 2 | NM_176872 | -5,6759 | <0.05 |
| Bt.28472.1.S1_at | OLFML3 | olfactomedin-like 3 | NM_001075197 | -5,46562 | <0.05 |
| Bt.4345.1.S1_at | FAS | Fas (TNF receptor superfamily, member 6) | NM_174662 | -5,14779 | <0.05 |
| Bt.23178.1.S1_at | DCN | decorin | NM_173906 | -5,01376 | <0.05 |
| Bt.15886.1.S1_at | ACSL5 | acyl-CoA synthetase long-chain family member 5 | NM_001075650 | -5,01375 | <0.05 |
| Bt.23216.1.S1_at | ENPP2 | ectonucleotide pyrophosphatase/phosphodiesterase 2 | NM_001080293 | -5,00938 | <0.05 |
| Bt.22526.1.S1_at | HSPB8 | heat shock 22kDa protein 8 | NM_001014955 | -4,94392 | <0.05 |
| Bt.4209.2.S1_a_at | C3 | complement component 3 | NM_001040469 | -4,93862 | <0.05 |
| Bt.4057.1.S1_at | MYH10 | myosin, heavy chain 10, non-muscle | NM_174834 | -4,7907 | <0.05 |
| Bt.4635.1.S1_at | CNN2 | calponin 2 | NM_001035420 | -4,68607 | <0.05 |
| Bt.5021.1.S1_at | FBN1 | fibrillin 1 | NM_174053 | -4,61867 | <0.05 |
| Bt.3011.1.A1_at | THBS3 | thrombospondin 3 | NM_001101839 | -4,37222 | <0.05 |
| Bt.20035.2.A1_at | CXXC5 | CXXC finger 5 | NM_001038176 | -4,20399 | <0.05 |
| Bt.23672.1.A1_at | ROBO1 | roundabout, axon guidance receptor, homolog 1 (Drosophila) | XM_616959 | -4,18341 | <0.05 |
| Bt.8124.1.S1_at | COL1A2 | collagen, type I, alpha 2 | NM_174520 | -4,02379 | <0.05 |
| Bt.23318.1.S1_at | COL3A1 | collagen, type III, alpha 1 | NM_001076831 | -3,97877 | <0.05 |
| Bt.4895.1.S1_at | CCND2 | cyclin D2 | NM_001076372 | -3,94196 | <0.05 |
| Bt.4915.1.S1_at | COTL1 | coactosin-like 1 (Dictyostelium) | NM_001046593 | -3,77418 | <0.05 |
| Bt.12906.1.S1_at | GSN | gelsolin (amyloidosis, Finnish type) | NM_001034627 /// NM_001113284 | -3,76648 | <0.05 |
| Bt.5250.1.S1_at | MFGE8 | milk fat globule-EGF factor 8 protein | NM_176610 | -3,65408 | <0.05 |
| Bt.12722.1.A1_at | ANG /// RNASE4 | angiogenin, ribonuclease, RNase A family, 5 /// ribonuclease, RNase A family, 4 | NM_001040590 /// NM_001078144 | -3,61966 | <0.05 |
| Bt.4733.1.S1_at | PHGDH | phosphoglycerate dehydrogenase | NM_001035017 | -3,61018 | <0.05 |
| Bt.7190.1.S1_at | CYP11A1 | cytochrome P450, family 11, subfamily A, polypeptide 1 | NM_176644 | -3,60636 | <0.05 |
| Bt.13273.1.S1_at | SOCS2 | suppressor of cytokine signaling 2 | NM_177523 | -3,59356 | <0.05 |
| Bt.13975.1.S1_a_at | APOD | apolipoprotein D | NM_001076301 | -3,56425 | <0.05 |
| Bt.9933.1.S1_at | PTPRS | protein tyrosine phosphatase, receptor type, S | XM_617648 | -3,54354 | <0.05 |
| Bt.5302.1.S1_a_at | MRVI1 | murine retrovirus integration site 1 homolog | NM_174392 /// NM_194465 | -3,52755 | <0.05 |
| Bt.13198.1.S1_at | SIRPA | signal-regulatory protein alpha | NM_175788 | -3,42738 | <0.05 |
| Bt.27361.1.A1_at | TNFRSF6B | Tumor necrosis factor receptor superfamily, member 6b, decoy | NM_001101306 | -3,3654 | <0.05 |
| Bt.23000.2.S1_a_at | TBC1D2B | TBC1 domain family, member 2B | XM_616041 | -3,33138 | <0.05 |
| Bt.28704.1.S1_at | PLSCR4 | phospholipid scramblase 4 | NM_001081732 | -3,26416 | <0.05 |
| Bt.24864.1.S1_a_at | TFPI | tissue factor pathway inhibitor (lipoprotein-associated coagulation inhibitor) | XM_585593 | -3,25591 | <0.05 |
| Bt.3007.1.S1_at | TCFL5 | transcription factor-like 5 (basic helix-loop-helix) | XM_591493 | -3,18187 | <0.05 |
| Bt.2359.1.A1_at | FYN | FYN oncogene related to SRC, FGR, YES | NM_001077972 | -3,15178 | <0.05 |
| Bt.27854.1.S1_at | NFIL3 | nuclear factor, interleukin 3 regulated | NM_001075240 | -3,11725 | <0.05 |
| Bt.2441.1.S1_at | RTKN | rhotekin | NM_001034681 | -3,08736 | <0.05 |
| Bt.959.1.S1_at | FLNA | filamin A, alpha (actin binding protein 280) | XM_614269 | -3,03294 | <0.05 |
| Bt.4303.1.S1_at | ADA | adenosine deaminase | NM_173887 | -2,95219 | <0.05 |
| Bt.13150.1.S1_at | EPOR | erythropoietin receptor | XM_583711 | -2,90952 | <0.05 |
| Bt.663.1.S1_at | PALLD | palladin, cytoskeletal associated protein | XM_869983 | -2,90886 | <0.05 |
| Bt.5224.1.S1_at | DPYSL2 | dihydropyrimidinase-like 2 | NM_001076000 | -2,7854 | <0.05 |
| Bt.7871.1.S1_at | PDLIM2 | PDZ and LIM domain 2 (mystique) | NM_001034430 | -2,76215 | <0.05 |
| Bt.23502.1.S1_at | RAMP2 | receptor (G protein-coupled) activity modifying protein 2 | NM_001098860 | -2,68308 | <0.05 |
| Bt.1424.1.S1_at | KLF4 | Kruppel-like factor 4 (gut) | NM_001105385 | -2,60997 | <0.05 |
| Bt.4251.1.S1_at | IFNGR2 | interferon gamma receptor 2 (interferon gamma transducer 1) | XM_592804 | -2,60141 | <0.05 |
| Bt.10777.1.S1_at | FOXP1 | Forkhead box P1 | NM_001083689 | -2,58041 | <0.05 |
| Bt.27883.1.S1_at | EFS | embryonal Fyn-associated substrate | NM_001098910 | -2,51455 | <0.05 |
| Bt.5366.1.S1_at | PROS1 | protein S (alpha) | NM_174438 | -2,48968 | <0.05 |
| Bt.540.1.S1_at | PDE5A | phosphodiesterase 5A, cGMP-specific | NM_174417 | -2,46627 | <0.05 |
| Bt.27084.1.S1_at | SEMA3C | Sema domain, immunoglobulin domain (Ig), short basic domain, secreted, (semaphorin) 3C | NM_001101082 | -2,43815 | <0.05 |
| Bt.12506.1.S1_at | SERPINE2 | serpin peptidase inhibitor, clade E (nexin, plasminogen activator inhibitor type | NM_174669 | -2,39954 | <0.05 |
| Bt.6630.1.S1_at | CNN1 | calponin 1, basic, smooth muscle | NM_001046379 | -2,37235 | <0.05 |
| Bt.24902.1.S1_at | TPBG | Trophoblast glycoprotein | XM_593502 | -2,36606 | <0.05 |
| Bt.12912.1.S1_at | COL4A1 | collagen, type IV, alpha 1 | XM_580317 | -2,34826 | <0.05 |
| Bt.2046.1.S1_at | SRPX | sushi-repeat-containing protein, X-linked | NM_001040489 | -2,32343 | <0.05 |
| Bt.21766.1.A1_at | MLF1 | Myeloid leukemia factor 1 | NM_001038158 | -2,28594 | <0.05 |
| Bt.22045.1.S1_at | STEAP2 | six transmembrane epithelial antigen of the prostate 2 | NM_001077847 | -2,23735 | <0.05 |
| Bt.21085.1.S1_at | ITSN1 | Intersectin 1 (SH3 domain protein) | XM_001249464 | -2,23185 | <0.05 |
| Bt.17491.1.A1_at | CAMK1D | calcium/calmodulin-dependent protein kinase ID | XM_605252 | -2,22979 | <0.05 |
| Bt.5493.1.S1_at | S100A4 | S100 calcium binding protein A4 | NM_174595 | -2,17684 | <0.05 |
| Bt.4718.1.S1_at | PCTP | phosphatidylcholine transfer protein | NM_174835 | -2,15998 | <0.05 |
| Bt.3323.1.S1_at | PLXNB2 | plexin B2 | XM_584919 | -2,15325 | <0.05 |
| Bt.22842.1.S1_at | TGFB1I1 | transforming growth factor beta 1 induced transcript 1 | NM_001035313 | -2,13053 | <0.05 |
| Bt.5226.1.S1_at | SFRP1 | secreted frizzled-related protein 1 | NM_174460 | -2,05562 | <0.05 |
| Bt.7955.1.S1_at | PCOLCE | procollagen C-endopeptidase enhancer | NM_001045888 | -1,94487 | <0.05 |
| Bt.5141.1.S2_at | B4GALT1 | UDP-Gal:betaGlcNAc beta 1,4- galactosyltransferase, polypeptide 1 | NM_177512 | -1,93807 | <0.05 |
| Bt.4557.1.S1_at | IFNAR1 | interferon, alpha; receptor | NM_174552 | -1,87705 | <0.05 |
| Bt.24073.1.S1_at | IL1R1 | interleukin 1 receptor, type I | XM_593695 | -1,69267 | <0.05 |
| Bt.17672.1.S1_at | ARHGEF2 | Rho/rac guanine nucleotide exchange factor (GEF) 2 | XM_001789027 /// XM_001789031 | -1,66436 | <0.05 |
| Bt.17772.1.S1_at | MDM2 | Mdm2 p53 binding protein homolog (mouse) | NM_001099107 | -1,6145 | <0.05 |

Significance of difference in gene expression between our cell populations was calculated by means of the Mann-Whitney test. The

bolded genes were chosen to be validated by immunohistochemistry.
