# Supplementary material for: Acquisition Order of Ras and p53 Gene Alterations Defines Distinct Adrenocortical Tumor Phenotypes
Source: PLoS Genet. 2012 May 10;8(5):e1002700. doi: 10.1371/journal.pgen.1002700 (PMC3349738; doi:10.1371/journal.pgen.1002700)
Supplement: Table S2 — Primers for QRT–PCR. (DOC) [file pgen.1002700.s004.doc]

| Genes | Sense | Anti-sense |
| --- | --- | --- |
| Sparc  LRIG1  TPD52  EGLN2  CCND1  RP-L27 | AGACATGTGACCTGGACAACGACA  TGAGATTTCGGGCACAATCGAGGA  AACAAGCCTGCTGGTGGTGATTTC  GGCGCATGTGTATGCTGTTCAGTT  AGCTGGTCCTGGTGAACAAACTCA  CGGCTATCGTGAAGAACATTG | AGGCATGGCTGTTAATCACGAGGT  TCTCTTGGCCACGGACTTGATCTT  AAGATGTGCTTGGATCTCGCTTGC  ACGAGGCACACAAAGATTGAAGGC  CGTGTTTGCGGATGATCTGCTTGT  CCTTGCGTTTGAGAGCAGGG |

Table S2: Primers for QRT-PCR
